# PSMA radioligand therapy for solid tumors other than prostate cancer: background, opportunities, challenges, and first clinical reports

**DOI:** 10.1007/s00259-021-05433-w

**Published:** 2021-06-12

**Authors:** M. J. M. Uijen, Y. H. W. Derks, R. I. J. Merkx, M. G. M. Schilham, J. Roosen, B. M. Privé, S. A. M. van Lith, C. M. L. van Herpen, M. Gotthardt, S. Heskamp, W. A. M. van Gemert, J. Nagarajah

**Affiliations:** 1 Department of Medical Oncology, Radboud Institute for Health Sciences, Radboud University Medical Center, Nijmegen, The Netherlands; 2 Department of Medical Imaging, Nuclear Medicine, Radboud Institute for Molecular Life Sciences, Radboud University Medical Center, Nijmegen, The Netherlands; 3 Department of Medical Imaging, Nuclear Medicine, Radboud Institute for Health Sciences, Radboud University Medical Center, Nijmegen, The Netherlands

**Keywords:** Solid tumors, Prostate-specific membrane antigen (PSMA), Radioligand therapy, PET/CT imaging, [^68^ Ga]Ga-PSMA, [^177^Lu]Lu-PSMA

## Abstract

In the past decade, a growing body of literature has reported promising results for prostate-specific membrane antigen (PSMA)-targeted radionuclide imaging and therapy in prostate cancer. First clinical studies evaluating the efficacy of [^177^Lu]Lu-PSMA radioligand therapy (PSMA-RLT) demonstrated favorable results in prostate cancer patients. [^177^Lu]Lu-PSMA is generally well tolerated due to its limited side effects. While PSMA is highly overexpressed in prostate cancer cells, varying degrees of PSMA expression have been reported in other malignancies as well, particularly in the tumor-associated neovasculature. Hence, it is anticipated that PSMA-RLT could be explored for other solid cancers. Here, we describe the current knowledge of PSMA expression in other solid cancers and define a perspective towards broader clinical implementation of PSMA-RLT. This review focuses specifically on salivary gland cancer, glioblastoma, thyroid cancer, renal cell carcinoma, hepatocellular carcinoma, lung cancer, and breast cancer. An overview of the (pre)clinical data on PSMA immunohistochemistry and PSMA PET/CT imaging is provided and summarized. Furthermore, the first clinical reports of non-prostate cancer patients treated with PSMA-RLT are described.

**Supplementary Information:**

The online version contains supplementary material available at 10.1007/s00259-021-05433-w.

## Introduction

Prostate-specific membrane antigen (PSMA) is a transmembrane protein that is encoded by the *FOLH1* (folate hydrolase 1) gene and was first discovered in prostate cancer cells [1]. Contrary to what the name suggests, PSMA is not only selective to prostate cancer cells [2, 3] but also expressed by neovascular endothelial cells of various cancers, including glioblastoma, kidney cancer, lung cancer, and breast cancer [4–6]. Preclinical data suggests that PSMA might be involved in cancer-related angiogenesis by degrading the extracellular matrix and participating in integrin signal transduction [7, 8].

To date, most clinical research on PSMA focuses on prostate cancer due to its exceptional high level of PSMA expression by tumor cells. Clinical studies evaluated the potential of PSMA imaging using radiolabeled PSMA antibodies (ProstaScint®, J591) and ligands (namely [^68^ Ga]Ga-PSMA-11 and [^18^F]F-PSMA-1007), mainly by positron emission tomography (PET), revealing higher tumor detection rates and higher tumor-to-background ratios compared to conventional imaging modalities [9–12]. Subsequently, PSMA targeting antibodies (J591) or ligands (PSMA-617 or PSMA-I&T) were labeled with therapeutic radionuclides such as lutetium-177 (^177^Lu) or actinium-225 (^225^Ac) respectively [13–19]. Driven by the favorable binding features and pharmacokinetics of ligands compared toavailable antibodies (low bone marrow toxicity due to faster clearance), PSMA ligands are currently the main focus for PSMA therapy in prostate cancer patients [13, 19]. Yet, comparative studies are still lacking. [^177^Lu]Lu-PSMA-617 has demonstrated promising results in prostate cancer patients in one prospective study, as well as several retrospective studies and compassionate use programs worldwide [14–16]. A phase II trial on [^177^Lu]Lu-PSMA in heavily pretreated progressive prostate cancer showed efficacy, i.e., a PSA decline > 50%, in 57% of patients and a progression-free survival of 7.6 months [13]. Moreover, a phase III registration trial (VISION) in advanced prostate cancer patients completed recruiting and final results are awaited at the end of 2021 (NCT03511664).

Since PSMA radioligand therapy (PSMA-RLT) demonstrated remarkable therapeutic efficacy in prostate cancer patients, the question arises whether PSMA-RLT could also achieve beneficial effects in other cancers expressing PSMA on the tumors cells themselves, or in the tumor-associated neovasculature.

The aim of this review is to assess which other solid cancers could potentially benefit from PSMA-RLT, based on PSMA expression levels and PSMA imaging data. Potential challenges and differences compared to prostate cancer are discussed. Additionally, the results of the first clinical reports of PSMA-RLT in solid tumors other than prostate cancer are presented.

## Methods

### Search strategy

**Fig. 1:**
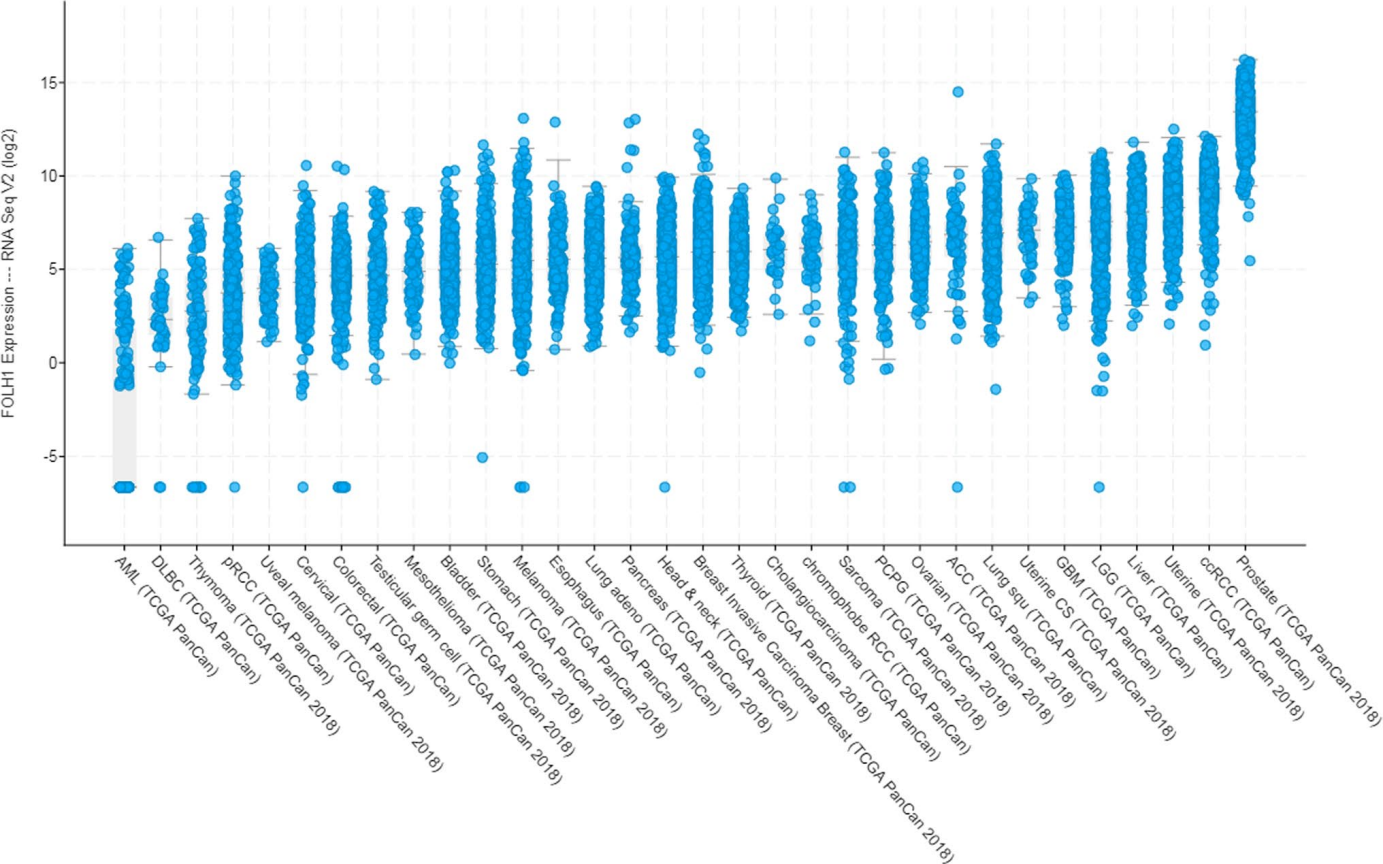
*FOLH1* expression levels of cancers included in TCGA PAN-CAN Atlas studies. This figure was adapted from cBioportal.org. Cancers are sorted based on median. Negative values are the result of the log(2) scale, where expression of 0 up to 1 in log(2) scaling results in negative values. Overall expression of mRNA in other cancers is considerably lower (log scale) than that in prostate cancer. All cancers show a large variation in *FOLH1* expression levels. Abbreviations: ACC adrenocortical carcinoma, AML acute myloid leukemia, DLBC diffuse large b-cell lymphoma, pRCC papillary renal cell carcinoma, RCC renal cell carcinoma, PCPG pheochromocytoma and paraganglioma, Uterine CS uterine carcinosarcoma, GBM glioblastoma multiforme, LGG lower grade glioma, ccRCC clear cell renal cell carcinoma

The selection of cancer types for this review was based on a combination of PSMA expression analysis and electronic library searches. First, the *FOLH1* gene expression levels (this gene encodes PSMA) of all cancers included in the TCG PanCancer Atlas were obtained from cBioPortal (Fig. 1) [20, 21]. Second, literature was searched by universal PubMed searches (see supplementary 1) for the fifteen cancers with the highest PSMA expression level on the PanCancer Atlas. Solely cancers with a substantial (> 20) amount of PubMed results were included in this review.

This resulted in the inclusion of glioblastoma, thyroid cancer, renal cell carcinoma, hepatocellular carcinoma, lung cancer, and breast cancer. Additionally, we included salivary gland cancer. Although this rare tumor entity is not included in the TCG PanCancer Atlas, several relevant PSMA-related studies were conducted for this cancer type.

For each type of cancer, the PubMed results were screened for papers or case reports which investigated PSMA expression levels through immunohistochemistry, PSMA imaging (e.g., PET/CT scans), or reports on PSMA-RLT. Both preclinical and clinical studies were included. The last search was performed on the 23rd of October 2020.

### PSMA immunohistochemistry

All articles reporting on PSMA immunohistochemistry (IHC) of the above-mentioned seven solid cancer types were included. No selection was made based on the type of antibody used for IHC staining, since there is no golden standard for PSMA IHC. Antibodies targeting the intracellular and extracellular domains of PSMA were included. A distinction was made between IHC staining on the tumor cells and the neovasculature. For each tumor type, the percentage of tumors which are PSMA positive on the IHC staining was described.

### PSMA PET/CT imaging

Although mRNA and PSMA IHC data provide relevant information on PSMA expression levels, in clinical practice, eligibility for PSMA-RLT in prostate cancer is based on in vivo tracer uptake revealed by PSMA PET/CT, semi-quantitatively expressed as standardized uptake values (SUV). According to the European Association of Nuclear Medicine (EANM) guideline based on the phase II trial on [^177^Lu]Lu-PSMA-617, the required maximum SUV (SUVmax) at dominant sites of tumor involvement should be at least 1.5-fold higher than the baseline mean SUV (SUVmean) of the liver on PET/CT (using renally excreted ligands such as [^68^ Ga]Ga-PSMA-11) to qualify for therapy [13, 22]. Therefore, we looked at the tumor/liver ratio; if this was not reported, we used a SUVmean of 4–8 for liver as reported in the literature [23, 24]. This suggests that a tumor uptake (SUVmax) of at least 12 might be considered sufficient to explore PSMA-RLT.

## Results

### Salivary gland cancer

Salivary gland cancer (SGC) is a rare and complex disease with an annual incidence of 2 per 100,000, consisting of 22 subtypes each with different clinical behaviors and prognoses [25, 26]. PSMA-related research has solely been conducted for adenoid cystic carcinoma (ACC) and salivary duct carcinoma (SDC).

Healthy salivary glands show high physiological tracer uptake on PSMA PET scans [9]. Interestingly, unlike prostate cancer cells, the uptake of PSMA ligands by the salivary glands does not seem to be completely mediated by PSMA; at least part of the uptake is aspecific [27, 28].

PSMA expression has been examined using IHC for both ACC and SDC in primary tumor material as well as metastases (details can be found in Table 1). The majority of ACC express PSMA on the tumor cells (91%—145/159 patients), while none of the tumors showed PSMA expression in the vasculature. In contrast, in SDC, the majority of the vessels express PSMA (90%—9/10), and some of the tumor cells express PSMA (40%—4/10) [29–33].

**Table 1:** Summary of PSMA expression and PSMA PET/CT imaging of seven different solid tumors

Cancer type	Subtype	PSMA expression Tumor cells IHC	PSMA expression Vasculature IHC	PSMA PET imaging	Proportion of patients possibly eligible for future PSMA-RLT studies
Salivary gland cancer	Adenoid cystic carcinoma	*Primary tumor* [29–33] N = 135 PSMA + : 93% (125/135) Positive cells: range: < 1–90% *Metastases * [29, 30, 33] *N* = 24 PSMA + : 83% (20/24) Positive cells: range: 5–100%	*Primary tumor* [29] *N* = 14 PSMA + : 0% (0/14) *Metastases * [29] *N* = 9 PSMA + : 0% (0/9)	*Primary/recurrent tumor *[29–31] *N* = 8 PET tracer uptake: 100% (8/8) SUVmax: range: 1.1 to 30.2* *Metastases* [29, 30, 32, 34, 35] *N* = 26 PET tracer uptake: 100% (26/26) SUVmax: range: 1.1 to 30.2*	93% of adenoid cystic carcinoma patients; tumor/liver ratio > 1 in 13/14 patients [29] Percentage of patients with tumor/liver ratio > 1.5 not reported
	Salivary duct carcinoma	*Primary tumor* [29] *N* = 9 PSMA + : 44% (4/9) Positive cells: range: < 1–50% *Metastases* [29] *N* = 1 PSMA + : 0% (0/1)	*Primary tumor *[29] *N* = 9 PSMA + : 89% (8/9) *Metastases *[29] *N* = 1 PSMA + : 100% (1/1)	*Primary/recurrent tumor* [29] *N* = 3 PET tracer uptake: 100% (3/3) SUVmax: range: 0.3 to 25.9* *Metastases* [29] *N* = 9 PET tracer uptake: 100% (3/3) SUVmax: range: 0.3 to 25.9*	40% of salivary duct carcinoma patients; tumor/liver ratio > 1 in 4/10 patients [29] Percentage of patients with tumor/liver ratio > 1.5 not reported
Glioblastoma	-	*Primary/recurrent tumor* [38–40] *N* = 8 PSMA + : 0% (0/8)	*Primary/recurrent tumor* [38–44] *N* = 128 PSMA + : 72% (92/128)	*Primary/recurrent tumor* [40, 42, 45–51, 53–55] *N* = 46 PET tracer uptake: 100% (46/46) SUVmax: range: 2.1 to 24.6	13% of glioblastoma patients tumor/liver ratio > 1.5 in 2/15 patients [54]
Thyroid cancer	Differentiated thyroid cancer	*Primary tumor* [58, 59] *N* = 209 PSMA + : 0% (0/209) *Metastases* [58] *N* = 9 PSMA + : 0% (0/9)	*Primary tumor* [58–60, 62] *N* = 258 PSMA + : 74% (192/258) *Metastases* [58] *N* = 9 PSMA + : 100% (9/9)	*Primary/recurrent tumor* [63, 64, 66, 74, 77, 108] *N* = 9 PET tracer uptake: 100% (9/9) SUVmax: range: 1.4 to 13.7 *Metastases* [63, 65, 67, 68, 73–75, 77] *N* = 29 PET tracer uptake: 100% (29/29) SUVmax: range: 0.9 to 101.8	Especially in metastatic disease, high tracer uptake has been reported [75] Some patients might be eligible for PSMA-RLT
	Anaplastic thyroid cancer	*Primary tumor* [58, 59] *N* = 15 PSMA + : 0% (0/15)	*Primary tumor* [58–60] *N* = 19 PSMA + : 63% (12/19)	*Primary/recurrent tumor* [72, 73] *N* = 2 PET tracer uptake: 100% (2/2) SUVmax: 6.0^‡^ *Metastases* [72] *N* = 1 PET tracer uptake: + SUVmax: NR	Insufficient data
	Medullary thyroid cancer	*Primary tumor* [59] *N* = 10 PSMA + : 0% (0/10)	*Primary tumor* [59–61] *N* = 126 PSMA + : 83% (104/126)	*Primary/recurrent tumor* [69, 71] *N* = 2 PET tracer uptake: 100% (2/2) SUVmax^‡^: 4.5 *Metastases* [70] *N* = 1 PET tracer uptake: + SUVmax: 19.7	Insufficient data Imaging data of one metastatic patient indicate sufficient tracer uptake of metastases
Renal cell carcinoma	Clear cell	*Primary tumor* [38, 132] *N* = 12 PSMA + : 0% (0/12)	*Primary tumor* [38, 81–83, 132, 133] *N* = 299 PSMA + : 79% (236/299) *Metastases* [134] *N* = 20 PSMA + : 75% (15/20)	*Primary/recurrent tumor* [85, 87, 88, 132, 135–140] *N* = 28 PET tracer uptake: 96% (27/28) SUVmax: range: 1.7 to 39.4 *Metastases* [85–89, 138–145] *N* = 36 PET tracer uptake: 89% (32/36) SUVmax: range: 0.9 to 48	In metastatic patients high tracer uptake has been reported. Some patients might be eligible for PSMA-RLT
	Papillary	*-*	*Primary tumor* [81–83, 133] *N* = 59 PSMA + : 27% (16/59)	*Primary/recurrent tumor* [85, 87, 89, 137] *N* = 4 PET tracer uptake: 50% (2/4) SUVmax: range: 3.6 to 5.1 *Metastases* [84] *N* = 3 PET tracer uptake: 67% (2/3) SUVmax: range: 1.8 to 4.1	Available data shows relatively low tracer uptake
	NS^†^	*-*	*-*	*Primary/recurrent tumor* [85, 137] *N* = 7 PET tracer uptake: 71% (5/7) SUVmax^‡^: 18.3 *Metastases* [84, 146] *N* = 3 PET tracer uptake: 67% (2/3) SUVmax: range: 0.5 to 6.2	Available data shows relatively low tracer uptake in metastatic patients
Hepatocellular carcinoma	-	*Primary tumor* [91–94] *N* = 282 PSMA + : 24% (69/282) Positive cells: NR	*Primary tumor* [91–94] *N* = 282 PSMA + : 83% (235/282)	*Primary/recurrent tumor* [92–102] *N* = 117 PET tracer uptake: 96% (112/117) SUVmax: range: 3.7 to 55.4 *Metastases* [94, 95, 99, 102] *N* = 16 PET tracer uptake: 100% (16/16) SUVmax: 2.2–21.3	100% of hepatocellular carcinoma patients; tumor/liver ratio > 1.5 in 15/15 patients [94]
Lung cancer	NSCLC—adenocarcinoma	*Primary tumor* [104, 105] *N* = 141 PSMA + : 15% (21/141) Positive cells: NR	*Primary tumor* [104, 105] *N* = 141 PSMA + : 45% (63/141)	*Primary tumor* [106, 107, 111] N = 3 PET tracer uptake: 100% (3/3) SUVmax: range: 4.8 to 5.6	Available PSMA imaging data indicates relatively low tracer uptake
	NSCLC—squamous cell carcinoma	*Primary tumor* [104, 105] *N* = 151 PSMA + : 19% (29/151) Positive cells: NR	*Primary tumor* [104, 105] *N* = 151 PSMA + : 64% (97/151)	-	Insufficient data
	NSCLC—large cell carcinoma	*Primary tumor* [104, 105] *N* = 70 PSMA + : 20% (14/70) Positive cells: NR	*Primary tumor* [104, 105] *N* = 70 PSMA + : 70% (49/70)	*-*	Insufficient data
	NSCLC—NS^†^	*Primary tumor* [38] *N* = 5 PSMA + : 0% (0/5)	*Primary tumor* [4, 38, 109] *N* = 13 PSMA + : 100% (13/13)	*Primary tumor* [108, 109] *N* = 9 PET tracer uptake: 100% (9/9) SUVmax: range: 3.7–7.0 *Metastases* [110] *N* = 1 PET tracer uptake: yes SUVmax: 4.4	Available PSMA imaging data indicates relatively low tracer uptake
	Small cell lung cancer	*Primary tumor *[104] *N* = 30 PSMA + : 0% (0/30)	*Primary tumor *[104] *N* = 30 PSMA + : 70% (21/30)	*-*	Insufficient data
Breast cancer	Invasive carcinoma of no special type	*Primary tumor* [38, 114] *N* = 56 PSMA + : 46% (26/56) Positive cells: NR	*Primary tumor* [38, 114, 118, 133] *N* = 312 PSMA + : 67% (209/312)	*Primary/recurrent tumor* [147, 148] *N* = 2 PET tracer uptake: 100% (2/2) SUVmax: range: 3.2 to 9.7	Insufficient data Available PSMA imaging data indicates relatively low tracer uptake
	Invasive lobular carcinoma	*Primary tumor *[38] *N* = 1 PSMA + : -	*Primary tumor* [38, 118] *N* = 65 PSMA + : 42% (27/65)	*-*	Insufficient data
	NS^†^	*Primary tumor* [114] *N* = 17 PSMA + : 29% (5/17) Positive cells: NR *Metastases* [114] *N* = 12 PSMA + : 75% (9/12) Positive cells: NR	*Primary tumor* [4, 114, 149] *N* = 110 PSMA + : 70% (77/110) *Metastases* [114, 149] *N* = 23 PSMA + : 96% (22/23)	*Primary/recurrent tumor* [116, 150] *N* = 14 PET tracer uptake: 57% (8/14) SUVmax: range: NR Mean SUVmax^¶^: 2.45 *Metastases* [116, 130, 131, 150–152] *N* = 19 PET tracer uptake: 89% (17/19) SUVmax: range: NR Mean SUVmax^¶^: 6.86	Available PSMA imaging data indicates relatively low tracer uptake

Abbreviations: IHC: immunohistochemistry, N: number of patients, NR: not reported, NS: not specified, NSCLC: non-small-cell lung cancer, PSMA: prostate specific membrane antigen, RLT: radioligand therapy, SUVmax: maximum standardized uptake value

*This study did not report separate SUVmax ranges for local recurrences or distant metastases. In ACC patients SUVmax ranged from 1.1 to 30.2 and in SDC patients SUVmax ranged from 0.3 to 25.9

†Some studies did not further specify the histology or outcomes were not separately reported for each histology

‡Some studies did not report SUVmax values, therefore only reported SUVmax values are presented in this table, but no range could be presented

¶Because SUVmax range could not be reported, the mean SUVmax of the study of Sathekge et al. was reported as an alternative indication of SUVmax values

**Fig. 2:**
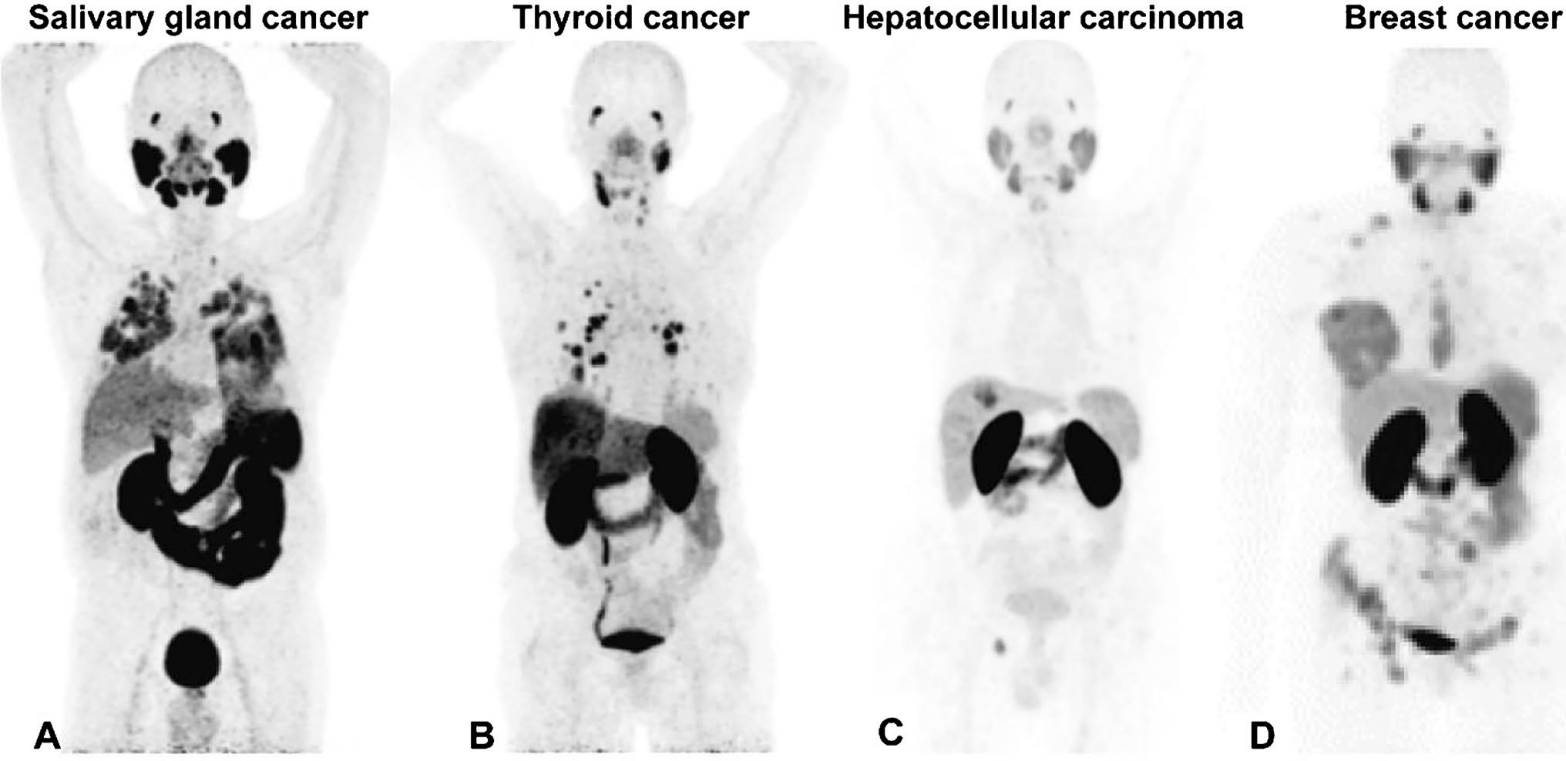
Four example PSMA PET/CT whole-body images of patients with salivary gland cancer, thyroid cancer, hepatocellular carcinoma, and breast cancer. **A** Patient with adenoid cystic carcinoma (salivary gland cancer) showing PSMA ligand uptake in lung metastases with a mean SUVmax of 10.0 and tumor-to-liver ratio of 2.5. **B** Patient with papillary thyroid carcinoma where PSMA PET/CT showed medium–high PSMA uptake in pulmonary metastases (median SUVmax 8.0). Additionally, new hotspots were seen on PSMA PET/CT (compared to [^18^F]FDG PET) in the left cervical lymph nodes (SUVmax 3.33) and liver (SUVmax 7.2). **C** Patient with hepatocellular carcinoma showing focal uptake with an SUVmax of 17.6 and tumor-to-liver ratio of 4.0, as well as a tiny lesion in the cutting line with an SUVmax of 8.4. **D** Patient with breast cancer where PSMA PET/CT imaging demonstrated multiple osseous metastasis and a primary right breast cancer. Patient A was originally published in van Boxtel et al., [^68^ Ga]Ga-PSMA-HBED-CC PET/CT imaging for adenoid cystic carcinoma and salivary duct carcinoma: a phase 2 imaging study, *Theranostics* 2020, Ivyspring International Publisher© [33]. Patient B was originally published in de Vries et al., [^68^ Ga]Ga-PSMA PET/CT in radioactive iodine-refractory differentiated thyroid cancer and first treatment results with [^177^Lu]Lu-PSMA-617. *EJNMMI Research* 2020, Springer Nature© [57]. Patient C was originally published in Kunikowska et al., [^68^ Ga]Ga-Prostate-Specific Membrane Antigen PET/CT: a novel method for imaging patients with hepatocellular carcinoma. *Eur J Nucl Med Mol Imaging*, Copyright 2020, Spinger Nature© [99]. Patient D was originally published in Sathekge et al., [^68^ Ga]Ga-PSMA-HBED-CC PET imaging in breast carcinoma patients. *Eur J Nucl Med Mol Imaging*, 2017, Springer Nature© [122]. These PSMA PET/CT images of four example patients were reprinted from open access articles distributed under the terms of the Creative Commons Attribution 4.0 International License (http://creativecommons.org/licenses/by/4.0/)

PSMA tracer uptake in ACC, as visualized with PSMA PET/CT, was first described in several case reports. Some patients demonstrated high PSMA uptake (SUVmax: 23.3) in metastatic lesions, compared to other patients who only showed low to modest tracer uptake in the tumor cells (SUVmax: 1.2) [31, 32, 34, 35]. This variation in uptake was confirmed in larger studies (van Boxtel et al. also included SDC patients), also describing a large variation of PSMA tracer uptake between patients (Fig. 2) [29, 30]. Even within a patient, a relatively large inter-metastatic variation in tracer uptake was detected [29]. van Boxtel et al. reported a tumor/parotid ratio, which was < 1 in the vast majority of cases [29]. Therefore, PSMA PET/CT imaging might be of limited value for detecting primary tumors or local recurrences, but could be useful for detecting lymph node or distant metastases. Overall, SUVmax values ranged from 1.1 to 30.2 in ACC patients and from 0.3 to 25.9 in SDC patients. This suggests that PSMA-RLT might be of interest for a subset of salivary gland cancer patients, since some patients showed lesions with a SUVmax > 12.

Regarding clinical studies on PSMA-RLT (Table 2), one patient with stage IV ACC received a single dose of [^177^Lu]Lu-PSMA (7.5 GBq) [34]. Treatment was well tolerated with no side effects and some pain relief was reported. Whole-body [^177^Lu]Lu-PSMA SPECT/CT imaging after therapy showed intense uptake in the metastases. A planned second cycle of [^177^Lu]Lu-PSMA was canceled due to malignancy-induced hypercalcemia, and the patient deceased soon after. Another study stated that one ACC patient was undergoing [^177^Lu]Lu-PSMA treatment, but details on the dose, toxicity, and therapeutic effect were not reported [30]. Currently, a prospective phase II pilot study of [^177^Lu]Lu-PSMA-I&T for ACC and SDC patients is recruiting (NCT04291300) offering a maximum of four cycles containing 7.4 GBq, every 6 weeks.

**Table 2:** Clinical reports and studies on PSMA-RLT in seven different solid tumors

Cancer	Subtype	Author, year	Number of patients	Radioligand	Injected activity, number of cycles	Efficacy*	Dosimetry	Comments
Salivary gland cancer	Adenoid cystic carcinoma	Klein Nulent et al. 2017. [30]	N = 1	[^177^Lu]Lu-PSMA-617	NR	NR	NR	Article mentions that one patient was undergoing [^177^Lu]Lu-PSMA treatment. But further details have not been reported
	Adenoid cystic carcinoma	Simsek et al. 2019. [34]	N = 1	[^177^Lu]Lu-PSMA	7.5 GBq 1 cycle	Pain reduction	Scan after 24 h, showed intense uptake of metastases	PSMA ligand not specified Second dose was intended but canceled to malignancy-induced hypercalcemia
	Adenoid cystic carcinoma and Salivary duct carcinoma	Study protocol: recruiting	Intended:N = 10	[^177^Lu]Lu-PSMA-I&T	7.4 GBq 2–4 cycles	NA	Will be performed after 1 h, 24 h, 48 h, 72 h and 7d	Clinical study: NCT04291300 Recruiting
Glioblastoma	-	Kunikowska et al. 2020. [53]	N = 1	[^177^Lu]Lu-PSMA-617	8.4 GBq 1 cycle	NR	Scans after 3 h, 24 h, 48 h, 7d and 14 d. Calculated tumor absorbed dose: 14.07 Gy	There were no efficacy related outcomes reported
	-	Kumar et al. 2020. [55]	N = 1	[^177^Lu]Lu-PSMA-617	3.7 GBq 3 cycles	- improvement performance status - symptom improvement - tumor reduction: from 17 mL to 5.4 mL	NR	
Thyroid cancer	Papillary thyroid carcinoma	De Vries et al. 2020. [77]	N = 2	[^177^Lu]Lu-PSMA-617	6 GBq 2 cycles	**Patient 1:** Partial temporary response of lung and liver metastases PFS: 7 months **Patient 2:** No response	NR	Both patients were heavily pretreated
	Radioactive iodine-refractory differentiated thyroid carcinoma	Assadi et al. 2019 [76]	N = 1	[^177^Lu]Lu-PSMA	7.4 GBq 1 cycle	NR	NR	PSMA ligand not specified Patient deceased 2 weeks post-therapy of sudden cardiac arrest
Renal cell carcinoma	-	-	-	-	-	-	-	
Hepatocellular carcinoma	-	Hirmas et al. 2021 [102]	N = 2	[^177^Lu]Lu-PSMA-617	5.9–6.2 GBq 1 cycle	NR	Intra-therapeutic SPECT/CT based dosimetry revealed low tumor radiation dose	Treatment was discontinued in both patients after low radiation doses based on SPECT/CT dosimetry
Lung cancer	-	-	-	-	-	-	-	
Breast cancer	Unknown, triple negative	Tolkach et al. 2018	N = 1	[^177^Lu]Lu-PSMA	7.5 GBq 2 cycles	No response	NR	PSMA ligand not specified Treatment was well tolerated, no side effects. Clinical follow-up showed severe progress after the second cycle, so no further cycles applied

Abbreviations: NA not applicable, NR not reported, h hours, d days, RLT radioligand therapy, PFS progression-free survival, PR partial response

*Efficacy: this included any of the following: objective or subjective response, progression-free survival, overall survival, quality of life

### Glioblastoma

Glioblastoma is the most frequently occurring type of brain cancer, with an annual incidence of 5 per 100,000 and is highly aggressive [36]. Glioblastomas are known to be highly vascularized tumors [37].

The first reports on immunohistochemical staining in glioblastoma tumors observed PSMA expression only in the neovasculature and not in the tumor cells [38–40]. Therefore, subsequent IHC studies primarily focused on the PSMA expression of the neovasculature [41–44]. Overall, 72% (92/128) of the glioblastoma tumors express PSMA in the neovasculature (Table 1). Two reports also quantified the vasculature staining by scoring the percentage of PSMA-positive vessels and staining intensity [41, 44]. Wernicke et al. [41] reported that in 69% of the tumors > 50% of the vessels were PSMA positive, while this was only the case in 32% of the tumors in Mahzouni et al. [44].

PSMA ligand uptake by glioblastoma tumors has been observed with different diagnostic radiotracers [40, 42, 45–51]. Bertagna et al. previously published a systematic review with a focus on the possible diagnostic role of PSMA PET/CT imaging, including most of these studies [52]. They concluded that glioblastomas are PSMA-avid tumors and that PSMA PET/CT imaging could be a useful diagnostic tool in glioblastoma. Articles published since then are in line with these conclusions [49–51, 53–55]. Regarding the diagnostic value, a major advantage of PSMA PET/CT imaging over [^18^F]FDG PET/CT imaging is the lower background uptake, since normal brain parenchyma shows physiological [^18^F]FDG uptake but no physiological PSMA uptake. In glioblastoma, [^18^F]FLT PET/CT is regularly performed, but no studies comparing this tracer with PSMA PET/CT are known. Overall, SUVmax in glioblastoma ranged between 2.1 and 24.6. Kunikowksa et al. reported tumor/liver ratios after [^68^ Ga]Ga-PSMA-11 PET [54]; 40% (6/15 patients) of the glioblastoma patients showed a tumor/liver ratio > 1, and 13% (2/15) had a tumor/liver ratio > 1.5. This suggests that at least part of the glioblastoma patients might have sufficient uptake to be considered for PSMA-RLT.

Kunikowska et al. published the first case report of PSMA-RLT in a glioblastoma patient (Table 2) [53]. This patient had a glioblastoma recurrence after prior treatments of surgery and chemo-radiotherapy. On [^68^ Ga]Ga-PSMA-11 PET, the patient had a SUVmax of 10.3 with homogenous tumor PSMA uptake. The patient received a single dose of 8.4 GBq [^177^Lu]Lu-PSMA. Although the report did not mention clinical outcome, intra-therapeutic serial SPECT imaging showed tracer accumulation in the tumor over time, with a calculated absorbed radiation dose of 14 Gy within the tumor. Recently, Kumar et al. reported about a patient who received PSMA-RLT which resulted in tumor shrinkage. This patient was pretreated with surgery, radiotherapy, and temozolomide before receiving 3 cycles of 3.7 GBq (every 8 weeks) of [^177^Lu]Lu-PSMA-617. Post-therapy MRI showed a partial response with a tumor shrinkage (from 18 to 5.4 mL) and importantly improvement of quality of life [55].

### Thyroid cancer

Thyroid cancer is an endocrine malignancy with an annual incidence of 2–6 per 100,000 [56]. The most common subtype is differentiated thyroid carcinoma (DTC), which includes papillary thyroid carcinoma (PTC) and follicular thyroid carcinoma (FTC) [57]. Other rare subtypes of thyroid cancer are medullary thyroid carcinoma (MTC) and anaplastic thyroid carcinoma (ATC) that have a dismal prognosis.

In thyroid cancer, the available literature did not report any PSMA expression on tumor cells itself in any of the subtypes [58, 59]. Immunohistochemical PSMA expression on the neovasculature has been examined for all thyroid carcinoma subtypes; details are shown in Table 1 [58–62]. Overall, PSMA expression in the neovasculature was observed in PTC (61%—134/220 patients), FTC (56%—43/77), MTC (83%—104/126), and ATC (63%—12/19). Of the PTC and FTC tumors that became dedifferentiated (so-called radio-iodine (RAI)-refractory), neovascular PSMA expression was reported in 63% of tumors (15/24) [59]. Interestingly, Sollini et al. found that PSMA expression levels in DTC patients contributed to the prediction of tumor aggressiveness and patient outcome [62].

PSMA tracer uptake on PET/CT imaging of thyroid cancer has been described in several case reports and subsequent larger prospective studies (Fig. 2) [63–75]. Overall, in DTC patients, PSMA tracer uptake seemed to differ between primary/recurrent lesions and metastatic lesions. SUVmax of primary/recurrent tumors ranged between 1.4 and 13.7, whereas in metastatic lesion the SUVmax range was 0.9 to 101.8. Therefore, especially metastatic DTC patients might have sufficient tracer uptake to be eligible for PSMA-RLT. PSMA uptake in ATC and MTC patients was only described in few patients, with relatively low SUVmax values (primary tumor uptake 4.5–6) [69–73]. Of these, Arora et al. reported a tumor/liver ratio > 2 in some MTC patients, indicating the possible eligibility of these patients for PSMA-RLT [69].

Regarding PSMA-RLT, literature reports on three treated thyroid cancer patients (Table 2) [76, 77]. Assadi et al. treated a progressing metastatic RAI-refractory DTC patient with [^177^Lu]Lu-PSMA [76]. The patient previously received RAI therapy, sorafenib therapy for 6 months, and radioligand therapy targeting the somatostatin receptor using [^177^Lu]Lu-DOTATATE (1 cycle, 7.4 GBq). Thereafter, 1 cycle of 7.4 GBq [^177^Lu]Lu-PSMA was given. Post-therapy whole‑body SPECT imaging revealed higher uptake of [^177^Lu]Lu-PSMA compared with whole‑body SPECT imaging following [^177^Lu]Lu‑DOTATE treatment; PSMA-RLT therapy is therefore more likely to be effective in this patient. Two weeks after [^177^Lu]Lu-PSMA therapy, the patient deceased unexpectedly due to cardiac arrest. In the study of de Vries et al., five patients with RAI-refractory DTC underwent PSMA PET/CT to determine their eligibility for [^177^Lu]Lu-PSMA therapy [77]. Three patients were considered eligible for PSMA-RLT, of whom two were treated with 2 cycles of 6 GBq [^177^Lu]Lu-PSMA-617. One of the patients did not respond to therapy and showed disease progression on [^18^F]FDG PET/CT after 1 month. Interestingly, the second patient did have a partial response of lung and liver metastases on imaging, and a transient decrease of the tumor marker thyroglobulin from 17 to 9 μg/L. Seven months post treatment, disease progression was observed on imaging and the thyroglobulin level increased to 14 μg/L. Both of these DTC case reports did not report on side effects of PSMA-RLT [76, 77].

### Renal cell carcinoma

Renal cell carcinoma (RCC) has an incidence of 4.4 per 100,000 [78]. Renal tumors are divergent and their clinical behavior is highly dependent on the histological subtype [79]. Clear cell RCC is the most common subtype and accounts for the majority of kidney cancer-related deaths [80]. Importantly, pro-angiogenic factors (VEGF, PDGF) are strongly upregulated in clear cell RCC, leading to high vascularized tumors. Other frequently occurring RCC subtypes include papillary RCC and chromophobe RCC.

Regarding the PSMA expression in RCC, most research has been conducted for clear cell RCC and papillary RCC (Table 1). PSMA expression of primary renal neoplasms demonstrated an exclusive PSMA expression in the tumor-associated neovasculature [5]. This holds true for clear cell RCC, papillary RCC, chromophobe RCC, and oncocytoma. Clear cell RCC was found to have the highest percentage of PSMA-positive tumors and also the highest PSMA staining intensity. In contrast, transitional cell and angiomyolipoma showed no PSMA expression [81, 82]. Overall, seventy-nine percent (236/299 patients) of the primary clear cell RCC tumors showed positive PSMA staining, in contrast to 27% (16/59) in primary papillary RCC. In addition, in metastatic clear cell RCC, 75% (15/20) of the tumors showed PSMA expression in the neovasculature [81–83]. Spatz et al. presented the largest cohort, with 257 RCC patients (including papillary, clear cell, and chromophobe subtypes). Interestingly, this cohort related stronger PSMA expression patterns with high-grade and advanced tumors and increased staining intensity was associated with poorer overall survival [81].

The role of PSMA PET/CT imaging in RCC has yet to be defined, but its potential has been investigated in multiple clinical studies. Due to the highest PSMA expression in clear cell RCC, this subtype has gained the most interest for clinical application. This consensus is reinforced by a recent report that showed inconsistent detection of non-clear cell RCC lesions [84]. Several explorative studies showed heterogeneity of PSMA uptake in clear cell RCC lesions; in primary/recurrent tumors, SUVmax ranged from 1.7 to 39.4, and in metastatic lesions, a range between 0.9 and 48 was reported [85–87, 89]. Since some of the SUVmax values described in literature are above 12, a part of the patients might be considered for PSMA-RLT. This is supported by Siva et al. who also mentioned that [^177^Lu]Lu-PSMA treatment might be feasible in a part of the recurrent RCC patients based on the high PSMA tracer uptake [89].

To date, no RCC patients have been treated with PSMA-RLT according to literature.

### Hepatocellular carcinoma

Hepatocellular carcinoma (HCC) is the most frequent primary liver cancer with an incidence of 10.1 cases per 100,000 person-years [90].

Immunohistochemical PSMA expression has been examined in 282 primary HCC tissue samples (Table 1) [91–94]. Overall, PSMA expression was mostly observed in the tumor-associated neovasculature (83%—235/282), and it was associated with poor prognosis in patients with HCC [91]. Only one of the studies also identified some PSMA expression by the tumor parenchyma (41% of samples), in a canalicular pattern [93].

Regarding PSMA-RLT (Table 2), two HCC patients received PSMA-RLT [10]. Both patients were treated with one cycle of [^177^Lu]Lu-PSMA-617 (activity 5.9–6.2 GBq). Although the treatment was well tolerated, intra-therapeutic SPECT/CT-based dosimetry revealed disappointing radiation dosages. According to the authors, the PSMA-RLT dose was at least tenfold lower than typically achieved by one cycle of external beam radiation therapy for HCC. Therefore, PSMA-RLT was discontinued after one cycle for both patients.

### Lung cancer

The incidence of lung cancers varies largely between countries and differs between sexes. It ranges from < 10 to > 50 per 100,000 person-years [103]. Lung cancer is generally divided into small cell lung cancer (SCLC) or non-small cell lung cancer (NSCLC). NSCLC can be further classified into adenocarcinoma (most common form), squamous cell carcinoma, and large cell carcinoma [103].

In lung cancer, presence of PSMA is mainly observed on the neovasculature (Table 1), with expression levels in primary tumors ranging from 45% (63/141 patients) in adenocarcinoma to 70% in large cell carcinoma (49/70) and SCLC (21/30) [104, 105]. Positive staining of the tumor cells was shown by Wang et al. in all three subtypes of NSCLC, in approximately half of the cases [104]. However, this was not observed by Schmidt et al. where only a small fraction of the NSCLC cases were PSMA positive on the tumor cells (2–12%) [105]. PSMA expression on SCLC tumor cells has only been studied by Wang et al., who showed no PSMA expression [104].

All literature on PSMA tracer uptake by lung cancer derives from accidental findings of lung lesions on PSMA PET/CT scans in patients who received PSMA imaging for their prostate cancer [106–111]. The reported SUVmax of lung cancer ranged from 4.8 to 5.6 in lung adenocarcinoma (Table 1). In 9 patients with NSCLC (without further details on subtype), SUVmax ranged from 3.7 to 7.0. However, these lung cancers are identified due to their PSMA tracer uptake, and most likely PSMA-negative lung cancers have not been published. Therefore, it remains unclear which proportion of lung cancer patients show PSMA tracer uptake and to what extent (SUV values).

Case reports on PSMA-RLT for lung cancer patients were not identified.

### Breast cancer

Breast cancer is one of the most prevailing types of cancer, with an incidence of 128.5 per 100,000 woman per year [112]. The most common histopathological subtype, accounting for 75% of all breast cancers, is invasive carcinoma of no special type (IC-NST), formerly known as invasive ductal carcinoma [113]. The second most common type is invasive lobular carcinoma (ILC) (5–10%).

Immunohistochemical PSMA expression has been examined for both primary IC-NST and ILC tumors (Table 1). Overall, 67% (209/312 patients) of IC-NST tumors and 42% (27/65) of ILC tumors expressed PSMA in the neovasculature. Interestingly, Kasoha et al. also found weak to moderate PSMA expression on the tumor cells in 51% of IC-NST tumors, yet Chang et al. did not observe PSMA expression on the tumor cells in breast cancer [38, 114]. In metastatic breast cancer, PSMA expression was described in two reports; 96% (22/23) of these samples were positive for PSMA in the neovasculature [114, 115]. Remarkably, Wernicke et al. described that PSMA expression of all tumor metastases correlated with PSMA expression intensity of the primary tumor. They also found that both estrogen and progesterone receptor-negative tumors were more likely to have higher PSMA expression compared to hormone receptor-positive tumors [115].

Reports on PSMA PET/CT imaging mainly consisted of case reports (Fig. 2), which provided limited data on SUVmax or tumor/liver ratios. One article reported on PSMA imaging in 19 breast cancer patients (68% with IC-NST) [116]. The SUVmean was 2.5 ± 2.6 for primary or local recurrences (*n* = 13) and 3.2 ± 1.8 for involved lymph nodes (*n* = 15). Distant metastases (*n* = 53) showed a significantly higher SUVmean of 6.9 ± 5.7 compared to primary tumor/local recurrence (*p* = 0.04) and lymph node metastases (*p* = 0.011). SUVmean did not show a significant correlation with hormone receptor status; however, PSMA uptake increased with tumor grading and was more often seen in IC-NST compared to other histological subtypes. Based on the limited literature, the SUVmax values that have been reported in breast cancer are generally low (mean SUVmax: 2.5–6.9) suggesting limited potential for PSMA-RLT in breast cancer.

Interestingly, we found a preclinical PSMA-related study in breast cancer. This study investigated the potential of PSMA-RLT in breast cancer, showing that [^177^Lu]Lu-PSMA strongly impaired the vitality and angiogenic capacity of endothelial cells cultured in breast cancer conditioned medium [117]. Regarding the clinical application of PSMA-RLT (Table 2), a 38-year-old female with an aggressive triple-negative breast carcinoma, previously unresponsive to chemotherapy and bevacizumab, received [^177^Lu]Lu-PSMA-RLT (2 cycles 7.5 GBq) based on intense tumor tracer uptake on PSMA imaging (SUV not reported). Post-therapy SPECT imaging showed uptake in the tumor lesions and the treatment was well tolerated. However, severe disease progression was seen after the second treatment cycle and treatment was terminated [118]. No other case reports on PSMA-RLT in breast cancer were found.

## Discussion

Increasing evidence shows that PSMA-RLT is an effective treatment for prostate cancer patients with a favorable toxicity profile [13, 119]. Currently, PSMA-RLT is investigated in a phase III trial (VISION; NCT03511664). These promising results in prostate cancer in combination with literature showing PSMA expression and PSMA tracer uptake in other malignancies encouraged us to assess the potential role of PSMA-RLT for other solid cancer types [120]. We focused on PSMA expression, PSMA PET/CT tracer uptake, and results of clinical attempts of PSMA-RLT in seven different solid cancers.

Regarding PSMA immunohistochemistry, in the majority of the solid cancers included in this review, > 70% of the primary tumors showed PSMA expression on the tumor-associated neovasculature. Of all included primary tumors, medullary thyroid carcinomas and hepatocellular carcinomas most often expressed PSMA in the neovasculature. In contrast, in adenoid cystic carcinoma (subtype of salivary gland cancer) and papillary renal cell carcinoma, only few of the tumors showed PSMA-positive staining on the neovasculature. Interestingly, although most of the solid cancers did not express PSMA on the tumor cells, it was still observed in salivary gland tumors (especially in adenoid cystic carcinoma), and to minimal extent in hepatocellular, lung, and breast cancer tissue.

On PSMA PET/CT imaging, PSMA tracer uptake differed considerably between the solid cancers. In patients with salivary gland cancer, glioblastoma, thyroid cancer, hepatocellular cancer, and clear cell renal cell cancer, several patients showed relevant tumor tracer accumulation on PET imaging (SUVmax values ≥ 12). Extremely high SUVmax values up to 101.8 were seen in metastatic medullary thyroid carcinoma. On the other hand, in several types of lung cancer and breast cancer, tracer uptake was low to moderate at best (SUVmax < 10). Noteworthy, in solid cancers, intra-patient tumor heterogeneity was observed.

PSMA-RLT eligibility in prostate cancer is assessed through PSMA PET/CT imaging, with an eligibility cutoff tumor/liver ratio > 1.5 in [^68^ Ga]Ga-PSMA PET/CT according to the EANM guideline [22]. As elaborated on in the “Methods” section, for the aim of this review, we considered a tumor SUVmax of > 12 sufficient to potentially investigate PSMA-RLT. Bearing this in mind, we conclude that salivary gland cancer, glioblastoma, thyroid cancer (differentiated and medullary), hepatocellular carcinoma, and renal cell cancer (clear cell) are the most relevant tumors to further explore the potential of PSMA-RLT. In line with this, the first case reports on PSMA-RLT in patients other than prostate cancer included salivary gland cancer, glioblastoma, thyroid cancer, and hepatocellular carcinoma [30, 34, 53, 55, 76, 77, 102]. These nine heavily pretreated end-stage patients received 1–2 cycles of 5.9–8.4 GBq [^177^Lu]Lu-PSMA per cycle in compassionate use programs (Table 2). In some of these case reports, positive treatment outcomes were reported. In one salivary gland cancer patient, pain reduction was observed [34]. One thyroid cancer patient showed a partial response that lasted 7 months [77]. In a glioblastoma patient, tumor volume decreased upon PSMA-RLT [55]. Importantly, the treatment was generally well tolerated, with no or low-grade adverse events.

Despite the use of the same PSMA PET/CT-based eligibility criteria as in prostate cancer patients to assess possible PSMA-RLT application, there are essential differences between prostate cancer and the solid cancers included in this review.

First, in prostate cancer, PSMA is expressed on the tumor cells compared with the mainly neovascular expression in most of other solid cancers. Still, even if PSMA is solely expressed on the neovasculature of well-perfused tumors, PSMA-RLT could hypothetically induce a tumoricidal local radiation dose to the tumor cells due to the tissue range (2 mm) of beta particles emitted by radionuclides such as ^177^Lu [120]. Furthermore, radiation dosages to the neovasculature and tumor micro-environment might also lead to a harmful effect and induce secondary immune responses.

It has been speculated that PSMA expression solely on the neovasculature could result in a shortened tracer washout [53, 94], meaning that PSMA-RLT is not retained in the tumor for a longer time, resulting in a lower radiation dose to the tumor and less effective treatment. Yet, a case report on a glioblastoma patient treated with [^177^Lu]Lu-PSMA showed good tumor tracer retention on post-therapy imaging, resulting in a substantial tumor absorbed dose [53], and in another glioblastoma patient, [^177^Lu]Lu-PSMA treatment resulted in a decrease in tumor volume [55]. This suggests that a sufficient radiation dose might still be reached while PSMA expression is limited to the tumor vasculature. Nonetheless, dedicated studies including dosimetry are required to prove this.

Second, SUVmax values in other solid cancers are generally lower than the SUVmax (> 15–40) values in prostate cancer [121, 122]. This suggests that lower radiation doses in the tumor could be reached, likely leading to a lower fraction of patients responding to PSMA-RLT compared to prostate cancer patients.

Third, more intra-patient tumor heterogeneity in terms of PSMA expression is seen in other solid tumors compared to prostate cancer [123]. Supposedly, this is a result of the neovasculature versus tumor cell PSMA expression, as described above. To illustrate this, metastases with high neo-vascularization would have higher PSMA uptake compared to metastases with low neo-vascularization within a patient. However, even in patients with heterogenous PSMA tracer uptake, the bystander and abscopal effect known in radiation-oncology might lead to tumor responses in PSMA-negative tumors [124, 125].

Fourth, it has been long known that cancers vary in radio-sensitivity [126]. Prostate cancer is generally radiosensitive and external beam radiotherapy is effective in early-stage disease, which provided a good rationale for PSMA-RLT in metastatic prostate cancer [127]. In contrast, hepatocellular carcinoma, for example, is considered less radiosensitive [128, 129]. Therefore, some of the other solid cancers might require higher PSMA-RLT radiation doses, compared to prostate cancer, to achieve a clinically relevant response.

Based on this review, PSMA-RLT could potentially be investigated for certain solid cancers (e.g., salivary gland cancer, glioblastoma, thyroid cancer, liver cancer, and clear cell renal cell cancer). This has also been proposed for these cancers by other authors [29, 30, 46, 54, 76, 89, 95]. Sufficient PSMA uptake on PSMA PET/CT is a crucial parameter to consider therapy, and since a significant fraction of prostate cancer patients with high PSMA PET/CT tracer uptake do not respond to PSMA-RLT, other parameters, which are not clearly identified yet, obviously play also a relevant role. Hence, a good pre-selection of patients is crucial to apply this therapy in these patients in the future.

The current literature on PSMA uptake in cancers other than prostate cancer is scarce and prospective studies are rare. Therefore, it is not possible to draw firm, generalized conclusions. Furthermore, many of the papers included in this review are case reports. It is likely that patients with high PSMA uptake are reported, while negative results are less likely to be published, known as publication bias. Therefore, it may appear as if more tumors are PSMA avid, or have higher SUV values than is actually the case. In addition, relevant data such as PSMA tracer uptake (SUVmax) was frequently not reported [40, 49, 50, 64, 67, 69, 107, 130, 131].

### Future prospective

Currently, prospective PSMA PET/CT imaging studies in end-stage non-prostate cancer patients are lacking; consequently, reliable information on PSMA uptake is missing. In our opinion, prospective imaging studies are the key way towards exploring PSMA-RLT for non-prostate cancers, especially imaging studies in patients with advanced disease, as PSMA-RLT is likely to be explored in end-stage disease with limited other treatment options. This will provide essential information on PSMA uptake and enables estimating which proportion of patients could be eligible for PSMA-RLT.

Furthermore, when evaluating the potential of PSMA-RLT in other cancers, preclinical studies on the therapeutic effects of PSMA-RLT are currently lacking and would be advisable. These could provide relevant insight into the fundamental questions such as PSMA tracer retention in tumors where PSMA is limited to the neovasculature. Preclinical studies could also provide information on the sensitivity of non-prostate cancers to PSMA-RLT.

In a clinical setting, prospective therapeutic studies should be performed instead of single patient reports to prevent trial-and-error-based science. As a different PSMA localization (tumor cell surface in prostate cancer versus neovasculature in other solid cancers) might lead to different PSMA tracer kinetics, preferably these prospective studies should include multi-timepoint post-therapy imaging (dosimetry), to provide more information on PSMA tracer kinetics. Currently, a prospective therapeutic study in salivary gland cancer patients is recruiting (NCT04291300). Furthermore, pending the outcome of the pivotal trial for [^177^Lu]Lu-PSMA (VISION trial; NCT03511664) in prostate cancer patients, it is anticipated that with positive results the translation to other solid cancers may be accelerated.

## Conclusion

In summary, PSMA expression in solid cancers other than prostate cancer is primarily observed in the tumor neovasculature, with the exception of adenoid cystic carcinoma (subtype salivary gland cancer), where PSMA is expressed on the tumor cells. Although there is heterogeneity in PSMA expression and tracer uptake, a subset of patients with advanced salivary gland cancer, glioblastoma, thyroid cancer, hepatocellular carcinoma, and clear cell renal carcinoma show sufficient PSMA PET/CT tracer uptake in the tumor. These patients might potentially benefit from PSMA-RLT, so future research in this setting is encouraged. To date, ten patients with non-prostate solid cancers (salivary gland cancer, glioblastoma, thyroid cancer, hepatocellular carcinoma, and breast cancer) have been treated with PSMA-RLT and some beneficial effects were seen, making this an interesting topic for further exploration.

## Supplementary Information

Below is the link to the electronic supplementary material.

Supplementary file1 (PDF 420 KB)

## Abbreviations

PSMA: Prostate-specific membrane antigen
PSMA-RLT: Prostate-specific membrane antigen radioligand therapy
PET: Positron emission tomography
^68^Ga: Gallium-68
^177^Lu: Lutetium-177
SUV: Standardized uptake values
SPECT: Single-photon emission computed tomography
