# Supplementary material for: PSMA radioligand therapy for solid tumors other than prostate cancer: background, opportunities, challenges, and first clinical reports
Source: Eur J Nucl Med Mol Imaging. 2021 Jun 12;48(13):4350–68. doi: 10.1007/s00259-021-05433-w (PMC8566635; doi:10.1007/s00259-021-05433-w)
Supplement: Supplementary file 1 — Supplementary file1 (PDF 420 KB) [file 259_2021_5433_MOESM1_ESM.pdf]

Supplementary table 1: Search strategy PubMed

Exploratory searches PubMed (10-4-2020) used for inclusion (as described in methods).

**Type of cancer**<br>Search string	**Hits**
**Renal cell cancer**<br><br>(((PSMA[tiab] OR prostate specific membrane antigen[tiab] OR "FOLH1 protein, human" [Supplementary Concept]))) AND (((**renal cell**) AND (carcinoma*[tiab] OR cancer*[tiab] OR malignanc*[tiab] OR neoplasm*[tiab] OR tumor*[tiab] OR tumour*[tiab])))	**Hits: 105**
**Uterine cancer**<br><br>(((PSMA[tiab] OR prostate specific membrane antigen[tiab] OR "FOLH1 protein, human" [Supplementary Concept]))) AND (((**uterine OR endometrial**) AND (carcinoma*[tiab] OR cancer*[tiab] OR malignanc*[tiab] OR neoplasm*[tiab] OR tumor*[tiab] OR tumour*[tiab])))	Hits: 7
**Liver cancer**<br><br>(((PSMA[tiab] OR prostate specific membrane antigen[tiab] OR "FOLH1 protein, human" [Supplementary Concept]))) AND (((**liver**) AND (carcinoma*[tiab] OR cancer*[tiab] OR malignanc*[tiab] OR neoplasm*[tiab] OR tumor*[tiab] OR tumour*[tiab])))	**Hits: 175**
**Glioblastoma**<br><br>(((PSMA[tiab] OR prostate specific membrane antigen[tiab] OR "FOLH1 protein, human" [Supplementary Concept]))) AND ((((**glioblastoma**) AND (carcinoma*[tiab] OR cancer*[tiab] OR malignanc*[tiab] OR neoplasm*[tiab] OR tumor*[tiab] OR tumour*[tiab]))) OR **glioblastoma**)	**Hits: 23**
**Lung cancer**<br><br>(((PSMA[tiab] OR prostate specific membrane antigen[tiab] OR "FOLH1 protein, human" [Supplementary Concept]))) AND (((**lung**) AND (carcinoma*[tiab] OR cancer*[tiab] OR malignanc*[tiab] OR neoplasm*[tiab] OR tumor*[tiab] OR tumour*[tiab])))	**Hits: 114**
**Adrenocortical carcinoma**<br><br>(((PSMA[tiab] OR prostate specific membrane antigen[tiab] OR "FOLH1 protein, human" [Supplementary Concept]))) AND	Hits: 4

(((**adrenocortial**) AND (carcinoma*[tiab] OR cancer*[tiab] OR malignanc*[tiab] OR neoplasm*[tiab] OR tumor*[tiab] OR tumour*[tiab])))	
**Ovarian cancer**<br><br>(((PSMA[tiab] OR prostate specific membrane antigen[tiab] OR "FOLH1 protein, human" [Supplementary Concept]))) AND (((**ovarian**) AND (carcinoma*[tiab] OR cancer*[tiab] OR malignanc*[tiab] OR neoplasm*[tiab] OR tumor*[tiab] OR tumour*[tiab])))	Hits: 13
**Pheochromocytoma OR Paraganglioma**<br><br>(((PSMA[tiab] OR prostate specific membrane antigen[tiab] OR "FOLH1 protein, human" [Supplementary Concept]))) AND (**Pheochromocytoma** OR **Paraganglioma**)	Hits: 1
**Sarcoma**<br><br>(((PSMA[tiab] OR prostate specific membrane antigen[tiab] OR "FOLH1 protein, human" [Supplementary Concept]))) AND **sarcoma**	Hits: 15
**Cholangiocarcoma**<br><br>(((PSMA[tiab] OR prostate specific membrane antigen[tiab] OR "FOLH1 protein, human" [Supplementary Concept]))) AND ((((**cholangio**) AND (carcinoma*[tiab] OR cancer*[tiab] OR malignanc*[tiab] OR neoplasm*[tiab] OR tumor*[tiab] OR tumour*[tiab]))) OR **cholangiocarcinoma**)	Hits: 2
**Thyroid**<br><br>(((PSMA[tiab] OR prostate specific membrane antigen[tiab] OR "FOLH1 protein, human" [Supplementary Concept]))) AND (((**thyroid**) AND (carcinoma*[tiab] OR cancer*[tiab] OR malignanc*[tiab] OR neoplasm*[tiab] OR tumor*[tiab] OR tumour*[tiab])))	**Hits: 56**
**Breast cancer**<br><br>(((PSMA[tiab] OR prostate specific membrane antigen[tiab] OR "FOLH1 protein, human" [Supplementary Concept]))) AND (((**breast**) AND (carcinoma*[tiab] OR cancer*[tiab] OR malignanc*[tiab] OR neoplasm*[tiab] OR tumor*[tiab] OR tumour*[tiab])))	**Hits: 87**
**Salivary gland cancer**<br><br>(((PSMA[tiab] OR prostate specific membrane antigen[tiab] OR "FOLH1 protein, human" [Supplementary Concept]))) AND (((**Salivary gland**) AND (carcinoma*[tiab] OR cancer*[tiab]	**Hits: 93**

OR malignanc*[tiab] OR neoplasm*[tiab] OR tumor*[tiab] OR tumour*[tiab])))	

All cancer types with >20 hits on the exploratory searches were included in the review, which are highlighted in bold.

Final universal PubMed search strategy (23-10-2020)*

Search strategy PubMed	
**Pubmed**	
**#1**	(((((((((((PSMA[tiab]) OR prostate specific membrane antigen[tiab]) OR FOLH1[tiab]) OR FOLH[tiab]) OR folate hydrolase[tiab]) OR "FOLH1 protein, human" [Supplementary Concept])) OR Glutamaat carboxypeptidase II[tiab]) OR GCPII[tiab]) OR GCP2[tiab]) OR NAAG peptidase[tiab]) OR NAALADase I[tiab]) OR NAALDase1[tiab])
**#2**	((***type of cancer***) AND (carcinoma*[tiab] OR cancer*[tiab] OR malignanc*[tiab] OR neoplasm*[tiab] OR tumor*[tiab] OR tumour*[tiab]))
**#3**	#1 AND #2

*In addition to the articles identified through the elaborate search strategy, we included a recent article (published after de date of the final search on 23-10-2020). This article showed important PSMA imaging in hepatocellular carcinoma and reports on 2 patients treated with PSMA-radioligand therapy.

Study	mRNA expression	PSMA expression tumor cells IHC	PSMA expression vasculature IHC	PSMA PET imaging	J591 imaging	Proportion of patients possibly eligible for PSMA RLT	Comments
**Salivary gland cancer**							
**Subtype: adenoid cystic carcinoma**							
Klein Nulent, 2020 [1]	-	*Primary tumor*<br>**N=110**<br>PSMA+: 94% (103/110)<br>Median positive cells: 31% (IQR: 15-60%)<br><br>*Metastases*<br>**N=10**<br>PSMA+: 90% (9/10)<br>Median positive cells: 23% (IQR: 10-55%)	-	-	-	-	
Van Boxtel, 2020 [2]	-	*Primary tumor*<br>**N=14**<br>PSMA+: 79% (11/14)<br>median positive cells: 10% (range: <1-90%)<br><br>*Metastases*<br>**N=11**<br>PSMA+: 73% (8/11)<br>median positive cells: 5% (range: 5-80%)	*Primary tumor*<br>**N=14**<br>PSMA+: 0% (0/14)<br><br>*Metastases*<br>**N=9**<br>PSMA+: 0% (0/9)	*Primary/local recurrence*<br>**N=3**<br>PET uptake: 100% (3/3)<br>Mean SUVmax*: range: 4.8-13.8<br><br>*Metastases*<br>**N=14**<br>PET uptake: 100% (14/14)<br>Mean SUVmax*: range: 3.9-14.9	-	93%; tumor/liver-ratio >1: 13/14 patients	PET Radiotracer: ^{68}Ga-PSMA<br><br>*mean SUVmax per site of metastases were calculated.<br><br>Overall in ACC patients tumor SUVmax ranged from 1.1 to 30.2
Klein Nulent, 2017 [3]	-	*Primary tumor*<br>**N=9**<br>PSMA+: 100% (9/9)<br>median positive cells: 30% (range: 5-90%)	-	*Primary/local recurrence*<br>**N=4**<br>PET uptake: 100% (4/4)<br>Median SUVmax:	-	-	PET Radiotracer: ^{68}Ga-PSMA

Study	mRNA	PSMA IHC cells	PSMA IHC vessels	PSMA PET	J591 imaging	Relevant uptake for therapy	Comments
		Metastases<br>**N=3**<br>PSMA+: 100% (3/3)<br>median positive cells: 70% (range: 5-70%)		2.52<br>(IQR 2.41–5.95)<br><br>*Metastases*<br>**N=9**<br>PET uptake: 100% (9/9)<br>Median SUVmax: 4.01<br>(IQR 2.66–8.71)			
Koning, 2017 [4]	-	*Primary tumor*<br>**N=1**<br>PSMA+: yes<br>positive cells: 5%	-	*Primary/local recurrence*<br>**N=1**<br>PET uptake: yes<br>SUVmax: 23.25	-	-	PET Radiotracer: ^{68}Ga-PSMA
De Keizer, 2017 [5]	-	*Primary tumor*<br>**N=1**<br>PSMA+: yes<br>positive cells: NR	-	*Metastases*<br>**N=1**<br>PET uptake: yes<br>SUVmax: NR	-	-	
Lutje, 2016 [6]	-	-	-	*Metastases*<br>**N=1**<br>PET uptake: yes<br>SUVmax: NR	-	-	PET Radiotracer: ^{68}Ga-PSMA
Has Simsek, 2019 [7]	-	-	-	*Metastases*<br>**N=1**<br>PET uptake: yes<br>SUVmax: NR	-	-	PET Radiotracer: ^{68}Ga-PSMA
**Subtype: salivary duct carcinoma**							
Van Boxtel, 2020 [2]	-	*Primary tumor*<br>**N=9**<br>PSMA+: 44% (4/9)<br>median positive cells: 5% (range: <1%-50%)<br><br>*Metastases*<br>**N=1**<br>PSMA+: no	*Primary tumor*<br>**N=9**<br>PSMA+: 89% (8/9)<br><br>*Metastases*<br>**N=1**<br>PSMA+: yes	*Local recurrence*<br>**N=3**<br>PET uptake: 100% (3/3)<br>Mean SUVmax*: range: 4.0-16.8<br><br>*Metastases*<br>**N=9**	-	40%; tumor/liver-ratio >1 in 4/10 patients	PET Radiotracer: ^{68}Ga-PSMA<br><br>*mean SUVmax per site of metastases were calculated.<br><br>Overall in SDC patients tumor

Study	mRNA	PSMA IHC cells	PSMA IHC vessels	PSMA PET	J591 imaging	Relevant uptake for therapy	Comments
				PET uptake: 100% (9/9)<br>Mean SUVmax*: range: 1.8-14.3			SUVmax ranged from 0.3 to 25.9
**Glioblastoma**							
Chang, 1999 [8]	-	*Primary/local recurrence*<br>**N=1**<br>PSMA+: no	*Primary/local recurrence*<br>**N=1**<br>PSMA+: yes	-	-	-	
Salas Fragomeni, 2017 [9]	-	*Primary/local recurrence*<br>**N=2**<br>PSMA+: 0% (0/2)	*Primary/local recurrence*<br>**N=2**<br>PSMA+: 100% (2/2)	*Primary/local recurrence*<br>**N=2**<br>PET uptake: 100% (2/2)<br>SUVmax: NR	-	-	PET Radiotracer: [^{18}F]DCFPyL
Nomura, 2014 [10]	-	*Primary/local recurrence*<br>**N=5**<br>PSMA+: 0% (0/5)	*Primary/local recurrence*<br>**N=5**<br>PSMA+: 100% (5/5)	-	-	-	
Wernicke, 2011 [11]	-	-	*Primary/local recurrence*<br>**N=32**<br>PSMA+: 100% (32/32)	-	-	-	
Schwenck, 2015 [12]	-	-	*Primary/local recurrence*<br>**N=1**<br>PSMA+: yes	*Primary/local recurrence*<br>**N=1**<br>PET uptake: yes<br>SUVmax: : NR	-	-	PET radiotracer: ^{68}Ga-PSMA
Saffar, 2018 [13]	-	-	*Primary/local recurrence*<br>**N=27**<br>PSMA+: 41% (11/27)	-	-	-	
Mahzouni, 2019 [14]	-	-	*Primary/local recurrence*<br>**N=60**	-	-	-	

Study	mRNA	PSMA IHC cells	PSMA IHC vessels	PSMA PET	J591 imaging	Relevant uptake for therapy	Comments
			PSMA+: 67% (40/60)				
Sasikumar, 2017 [15]	-	-	-	*Primary/local recurrence*<br>**N=5**<br>PET uptake: 100% (5/5)<br>SUVmax: range 5.8-21.7	-	-	PET radiotracer: ^{68}Ga-PSMA
Kunikowska, 2018 [16]	-	-	-	*Primary/local recurrence*<br>**N=1**<br>PET uptake: yes<br>SUVmax: 23.7	-	-	PET radiotracer: ^{68}Ga-PSMA
Sasikumar, 2018 [17]	-	-	-	*Primary/local recurrence*<br>**N=10**<br>PET uptake: 100% (10/10)<br>SUVmax: was only reported for two patients: 4.07 and 22.3	-	-	PET radiotracer: ^{68}Ga-PSMA
Verma, 2019 [18]	-	-	-	*Primary/local recurrence*<br>**N=7**<br>PET uptake: 100% (7/7)<br>Mean SUVmax: 16.93 (range: 9.6-24.6)	-	-	PET radiotracer: ^{68}Ga-PSMA
Gupta, 2020 [19]	-	-	-	*Primary/local recurrence*<br>**N=1**<br>PET uptake: yes<br>SUVmax: NR	-	-	PET radiotracer: ^{68}Ga-PSMA

Study	mRNA	PSMA IHC cells	PSMA IHC vessels	PSMA PET	J591 imaging	Relevant uptake for therapy	Comments
Kunikowska, 2020 [20]	-	-	-	*Primary/local recurrence*<br>**N=1**<br>PET uptake: yes<br>SUVmax: 10.3	-	-	PET radiotracer: ^{68}Ga-PSMA
Kunikowska, 2020 [21]	-	-	-	*Primary/local recurrence*<br>**N=15**<br>PET uptake: 100% (15/15)<br>Median SUVmax: 6.5<br>(range: 2.1-14.3)	-	40%; tumor/liver ratio >1 in 6/15 patients<br><br>13%; tumor/liver ratio >1.5 in 2/15 patients	PET radiotracer: ^{68}Ga-PSMA
Marafi, 2020 [22]	-	-	-	*Primary/local recurrence*<br>**N=1**<br>PET uptake: yes<br>SUVmax: NR	-	-	PET radiotracer: ^{18}F-PSMA
Pilati, 2020 [23]	-	-	-	*Primary/local recurrence*<br>**N=1**<br>PET uptake: yes<br>SUVmax: NR	-	-	PET radiotracer: ^{68}Ga-PSMA
Kumar, 2020 [24]	-	-	-	*Local recurrence*<br>**N=1**<br>PET-uptake: yes<br>SUVmax: NR	-	-	
**Thyroid cancer**							
**Subtype: medullary thyroid carcinoma**							
Ciappuccini, 2019 [25]	-	-	-	*Primary/local recurrence*<br>**N=1**<br>PET uptake: yes<br>SUVmax: 4.5	-	-	PET radiotracer: ^{68}Ga-PSMA
Arora, 2018 [26]	-	-	-	*Primary/local recurrence*<br>**N=1**<br>PET uptake: yes	-	-	PET radiotracer: ^{68}Ga-PSMA

Study	mRNA	PSMA IHC cells	PSMA IHC vessels	PSMA PET	J591 imaging	Relevant uptake for therapy	Comments
				SUVmax: NR			
Arora, 2018 [27]	-	-	-	*Metastases*<br>**N=1**<br>PET uptake: yes<br>SUVmax: 19.7	-	N=1, tumor/liver ratio >2	PET radiotracer: ^{68}Ga-PSMA
Bychkov, 2017 [28]	-	*Primary tumor*<br>**N=10**<br>PSMA+: 0% (0/10)	*Primary tumor*<br>**N=10**<br>PSMA+: 40% (4/10)	-	-	-	
Heitkötter, 2018 [29]	-	-	*Primary tumor*<br>**N=12**<br>PSMA+: 33% (4/12)	-	-	-	
Lodewijk, 2018 [30]	-	-	*Primary tumor*<br>**N=104**<br>PSMA+: 92% (96/104)	-	-	-	
**Subtype: anaplastic thyroid carcinoma**							
Damle, 2018 [31]	-	-	-	*Primary/local recurrence*<br>**N=1**<br>PET uptake: yes<br>SUVmax: NR<br><br>*Metastases*<br>**N=1**<br>PET uptake: yes<br>SUVmax: NR	-	-	PET radiotracer: ^{68}Ga-PSMA
Moore, 2017 [32]	-	*Primary tumor*<br>**N=5**<br>PSMA+: 0% (0/5)	*Primary tumor*<br>**N=5**<br>PSMA+: 80% (4/5)	-	-	-	
Bychkov, 2017 [28]	-	*Primary tumor*<br>**N=10**<br>PSMA+: 0% (0/10)	*Primary tumor*<br>**N=10**<br>PSMA+: 40% (4/10)	-	-	-	
Heitkötter, 2018 [29]	-	-	*Primary tumor*<br>**N=4**<br>PSMA+: 100% (4/4)	-	-	-	
Lawhn-Heath, 2020 [33]	-	-	-	*Primary/local recurrence*<br>**N=1**			PET radiotracer: ^{68}Ga-PSMA

Study	mRNA	PSMA IHC cells	PSMA IHC vessels	PSMA PET	J591 imaging	Relevant uptake for therapy	Comments
				PET uptake: yes<br>SUVmax: 6.0			One other ATC patient had both a local recurrence and metastases, SUVmax was not separately reported per disease sites, but median SUVmax was 3.3
**Subtype: differentiated thyroid carcinoma (papillary + follicular + radioactive iodine refractory)**							
de Vries, 2020 [34]	-	-	-	*Primary/local recurrence*<br>**N=1**<br>PET uptake: yes<br>SUVmax: 1.38<br><br>*Metastases*<br>**N=5**<br>median SUVmax: depends on site of metastases.<br>SUVmax range: 0.9-10.6	-	60% (3/5); based on positive ^{68}Ga-PSMA PET.	PET radiotracer: ^{68}Ga-PSMA
Lengana, 2019 [35]	-	-	-	*Primary/local recurrence*<br>**N=1**<br>PET uptake: yes<br>SUVmax: NR<br><br>*Metastases*<br>**N=1**<br>PET uptake: yes<br>SUVmax: NR	-	-	PET radiotracer: ^{68}Ga-PSMA<br><br>Instead of SUXmax: SUVratio's were reported. These ratio's were calculated as a lesion to quadriceps muscle ratio using SUVmean.
Singh, 2018 [36]	-	-	-	*Primary/local recurrence*<br>**N=1**	-	-	PET radiotracer: ^{18}F-PSMA

Study	mRNA	PSMA IHC cells	PSMA IHC vessels	PSMA PET	J591 imaging	Relevant uptake for therapy	Comments
				PET uptake: yes<br>SUVmax: NR			
Lutje, 2017 [37]	-	-	-	*Primary/local recurrence*<br>**N=2**<br>PET uptake: yes<br>SUVmax: 4.3-13.7<br><br>*Metastases*<br>**N=5**<br>SUVmax range: 3.3-39.7	-	0%; tumor/liver ratio >1 in 0/5 patients	PET radiotracer: ^{68}Ga-PSMA<br><br>Higher SUVmax in FTC (N=3) compared to PTC (n=1).
Sager, 2016 [38]	-	-	-	*Primary/local recurrence*<br>**N=2**<br>PET uptake: yes<br>SUVmax: NR	-	-	PET radiotracer: ^{68}Ga-PSMA
Verma, 2018 [39]	-	-	-	*Metastases*<br>**N=10**<br>median SUVmax: 31.35 (range: 4.7-101.8)	-	Very high SUVmax are reported (up to 101.8)	PET radiotracer: ^{68}Ga-PSMA
Sasikumar, 2018 [40]	-	-	-	*Metastases*<br>**N=1**<br>PET uptake: yes<br>SUVmax: NR	-	-	PET radiotracer: ^{68}Ga-PSMA
Taywade, 2016 [41]	-	-	-	*Metastases*<br>**N=1**<br>PET uptake: yes<br>SUVmax: NR	-	-	PET radiotracer: ^{68}Ga-PSMA
Verburg, 2015 [42]	-	-	-	*Metastases*<br>**N=1**<br>PET uptake: yes<br>SUVmax: NR	-	-	PET radiotracer: ^{68}Ga-PSMA
Osman, 2017 [43]	-	-	-	*Primary*<br>**N=1**<br>PET uptake: yes<br>SUVmax: 1.9	-	-	PET radiotracer: ^{68}Ga-PSMA

Study	mRNA	PSMA IHC cells	PSMA IHC vessels	PSMA PET	J591 imaging	Relevant uptake for therapy	Comments
Moore, 2017 [32]	-	*Primary tumor*<br>Total: **N=37**<br><br>cPTC: **N=11**<br>PSMA+: 0% (0/11)<br><br>FvPTC: **N=9**<br>PSMA+: 0% (0/9)<br><br>FTC: **N=11**<br>PSMA+: 0% (0/11)<br><br>*Metastases*<br>**N=9**<br>PSMA+: 0% (0/9)	*Primary tumor*<br>Total: **N=37**<br><br>cPTC: **N=11**<br>PSMA+: 100% (11/11)<br><br>FvPTC: **N=9**<br>PSMA+: 78% (7/9)<br><br>FTC: **N=11**<br>PSMA+: 100% (11/11)<br><br>*Metastases*<br>**N=9**<br>PSMA+: 100% (9/9)	-	-	-	
Bychkov, 2017 [28]	-	*Primary tumor*<br>Total: **N = 172**<br><br>FTC: **N=52**<br>PSMA+: 0% (0/52)<br><br>PTC: **N=120**<br>PSMA+: 0% (0/120)<br><br>RAIR-DTC: **N=24**<br>PSMA+: 0% (0/24)	*Primary tumor*<br>Total: **N = 172**<br><br>FTC: **N=52**<br>PSMA+: 46% (24/52)<br><br>PTC: **N=120**<br>PSMA+: 51% (61/120)<br><br>RAIR-DTC: **N=24**<br>PSMA+: 63% (15/24)	-	-	-	
Heitkötter, 2018 [29]	-	-	*Primary tumor*<br>Total: **N=41**<br>FTC: **N=10**<br>PSMA+: 40% (4/10)<br><br>PTC: **N=31**	-	-	-	

Study	mRNA	PSMA IHC cells	PSMA IHC vessels	PSMA PET	J591 imaging	Relevant uptake for therapy	Comments
			PSMA+: 58% (18/31)				
Sollini, 2019 [44]	-	-	*Primary tumor*<br>Total: **N=53**<br>FTC: **N=4**<br>PSMA+: 100% (4/4)<br><br>PTC: **N=49**<br>PSMA+: 76% (37/49)	-	-	-	
Joshi, 2017, [45]	-	-	-	*Primary tumor*<br>**N=1** (PTC)<br>PET uptake: yes<br>SUVmax: NR	-	-	PET radiotracer: ^{68}Ga-PSMA
Lawhn-Heath, 2020 [33]	-	-	-	*Metastases*<br>**N=5**<br>PET uptake: yes<br>median SUVmax: 9.0 (range: 1.0-18.3)			PET radiotracer: ^{68}Ga-PSMA
**Renal cell cancer**							
**Subtype: clear cell**							
Chang, 1999 [8]	-	*Primary tumor*<br>**N=11**<br>PSMA+: 0% (0/11)	*Primary tumor*<br>**N=11**<br>PSMA+: 100% (11/11)	-	-	-	
Chang, 2001 [46]	-	-	*Metastases*<br>**N=20**<br>PSMA+: 75% (15/20)	-	-	-	In this study two anti-PSMA antibodies were used. Results of staining for the 7E11 antibody are reported here.
Kinoshita, 2006 [47]	-	-	*Primary tumor*§<br>**N=9**<br>PSMA+: 56% (5/9)	-	-	-	§Study did not mention if PSMA expression was observed in tumor

Study	mRNA	PSMA IHC cells	PSMA IHC vessels	PSMA PET	J591 imaging	Relevant uptake for therapy	Comments
							cells or neovasculature.
Baccalla, 2007 [48]	-	-	*Primary tumor* **N=21** PSMA+: 76% (16/21)	-	-	-	
Al-Hamadie, 2008 [49]	-	-	*Primary tumor* **N=30** PSMA+: 97% (29/30)	-	-	-	
Spatz, 2018 [50]	-	-	*Primary tumor* **N=228** PSMA+: 77% (175/228)	-	-	-	
Demirci, 2014 [51]	-	-	-	*Metastases* **N=1** Patient had 3 metastases PET uptake: yes Median SUVmax: 34.1 (range 28.3-35)	-	-	PET radiotracer: ^{68}Ga-PSMA
Rowe, 2015 [52]	-	-	-	*Metastases* **N=5** PET uptake: 100% (5/5) Median SUVmax: NR (range 1.6-19.3)	-	-	PET radiotracer: ^{18}F-DCFPyL
Rowe, 2016 [53]	-	-	-	*Metastases* **N=1** Patient had 2 metastases PET uptake: yes SUVmax = 2.7 and 3.2	-	-	PET radiotracer: ^{18}F-DCFPyL
Rhee, 2016 [54]	-	-	-	*Primary tumor* **N=10†**	-	-	PET radiotracer: ^{68}Ga-PSMA

Study	mRNA	PSMA IHC cells	PSMA IHC vessels	PSMA PET	J591 imaging	Relevant uptake for therapy	Comments
				PET uptake: 100% (10/10)<br>Mean SUVmax: 18.0 (3.7-36.5)<br><br>*Metastases*<br>**N=5‡**<br>PET uptake: unclear<br>Mean SUVmax: 19.5 (range 1.5-48)			†Mean SUVmax was only reported all included patients (N=10), 80% has clear cell, 10%: papillary and 10%: unclassified.<br><br>‡5/10 patients had metastases
Meyer, 2019 [55]	-	-	-	*Primary tumor*<br>**N=3**<br>PET uptake: 100% (3/3)<br>Median SUVmax: 9.6 (range 7.3-15.8)<br><br>*Metastases*<br>**N=14**<br>PET uptake: 75% (11/14)<br>Median SUVmax: 2.7 (range 0.9-38.5)	-	-	PET radiotracer: ^{18}F-DCFPyL<br><br>Study data indicates that the PSMA uptake of metastases is more heterogenous than of the primary lesions.
Sawicki, 2017 [56]	-	-	-	*Primary tumor*<br>**N=3**<br>PET uptake: 3/3 (100%)<br>Median SUVmax: 11.6 (range 1.7-27.2)<br><br>*Metastases*	-	-	PET radiotracer: ^{68}Ga-PSMA<br><br>For primary tumors the TBR of the SUVmax was 0.2 (range 0.02-0.7).<br><br>For metastases the TBR of the SUVmax

Study	mRNA	PSMA IHC cells	PSMA IHC vessels	PSMA PET	J591 imaging	Relevant uptake for therapy	Comments
				**N=2**<br>PET uptake: 2/2 (100%)<br>Mean SUVmax: 9.9 (range 3.4-25.6)			was 11.7 (range 4.4-28.1)
Milowsky, 2007 [57]	-	-	-	-	*Metastases*<br>**N=8**<br>75% (6/8) showed at least one area of known metastatic disease targeted by ^{111}In-J591	-	
Einspieler, 2016 [58]	-	-	-	*Metastases*<br>**N=1**<br>PET uptake: yes<br>SUVmax: NR	-	-	PET radiotracer: ^{68}Ga-PSMA
Rhee, 2016 [59]	-	*Primary tumor*<br>**N=1**<br>PSMA+: no	*Primary tumor*<br>**N=1**<br>PSMA+: yes	*Primary tumor*<br>**N=1**<br>PET uptake: yes<br>SUVmax: NR	-	-	PET radiotracer: ^{68}Ga-PSMA
Sasikumar, 2016 [60]	-	-	-	*Metastases*<br>**N=1**<br>PET uptake: yes<br>SUVmax: NR	-	-	PET radiotracer: ^{68}Ga-PSMA
Siva, 2017 [61]	-	-	-	*Recurrence/ metastases*<br>**N=7**<br>PET uptake: 86% (6/7)<br>Median SUVmax: 7.7 (range: 5.3-26.5)	-	-	PET radiotracer: ^{68}Ga-PSMA
Joshi, 2017 [45]	-	-	-	*Primary tumor*<br>**N=1**<br>PET uptake: yes	-	-	PET radiotracer: ^{68}Ga-PSMA

Study	mRNA	PSMA IHC cells	PSMA IHC vessels	PSMA PET	J591 imaging	Relevant uptake for therapy	Comments
				SUVmax: NR			
Zacho, 2017 [62]	-	-	-	*Metastases*<br>**N=1**<br>PET uptake: yes<br>SUVmax: NR	-	-	PET radiotracer: ^{68}Ga-PSMA
Gao, 2020 [63]	-	-	-	*Primary tumor*<br>N=36<br>PET uptake: unclear<br>SUVmax♣	-	-	PET radiotracer: ^{68}Ga-PSMA<br><br>♣: range of SUVmax not reported, of 2 patients SUVmax were reported: 6.2 and 39.4. Based on figure 2 SUVmax ranged from about 5-45.
Raveenthiran, 2019 [64]	-	-	-	*Primary tumor*<br>**N=8**<br>PET-uptake: 88% (7/8)<br>SUVmax: NR	-	-	PET radiotracer: ^{68}Ga-PSMA
Mittlemeier, 2020, [65]	-	-	-	*Primary/local recurrence*<br>**N=1**<br>PET-uptake: yes<br>SUVmax: NR<br><br>*Metastases*<br>**N=1**<br>PET-uptake: yes<br>SUVmax: NR	-	-	PET radiotracer: ^{18}F-PSMA
Marafi, 2020 [66]	-	-	-	*Primary tumor*<br>**N=1**<br>PET-uptake: yes<br>SUVmax: NR<br><br>*Metastases*<br>**N=1**	-	-	PET radiotracer: ^{18}F-PSMA

Study	mRNA	PSMA IHC cells	PSMA IHC vessels	PSMA PET	J591 imaging	Relevant uptake for therapy	Comments
				PET-uptake: yes<br>SUVmax: NR			
Chen, 2020 [67]	-	-	-	*Primary tumor*<br>**N=1**<br>PET-uptake: yes<br>SUVmax: 13.1<br><br>Metastases<br>**N=1**<br>SUVmax: range: 5.4-18.0	-	-	PET radiotracer: ^{68}Ga-PSMA<br><br>The patient multiple metastases, of 6 metastases, SUV max were reported.
**Subtype: papillary**							
Kinoshita, 2006 [47]	-	-	*Primary tumor*§<br>**N=2**<br>PSMA+: 100% (2/2)	-	-	-	§Study did not mention if PSMA expression was observed in tumor cells or neovasculature.
Al-Hamadie, 2008 [49]	-	-	*Primary tumor*<br>**N=15**<br>PSMA+: 73% (11/15)				
Baccalla, 2007 [48]	-	-	*Primary tumor*<br>**N=20**<br>PSMA+: 0% (0/20)	-	-	-	
Spatz, 2018 [50]	-	-	*Primary tumor*<br>**N=22**<br>PSMA+: 14% (3/22)	-	-	-	
Rhee, 2016 [54]	-	-	-	*Primary tumor*<br>**N=1**<br>PET uptake: yes<br>SUVmax: 3.6	-	-	PET radiotracer: ^{68}Ga-PSMA
Sawicki, 2017 [56]	-	-	-	*Primary tumor*<br>**N=1**<br>PET uptake: yes<br>SUVmax: 5.1	-	-	PET radiotracer: ^{68}Ga-PSMA
Milowsky, 2007 [57]	-	-	-	-	*Metastases*<br>**N=2**	-	

Study	mRNA	PSMA IHC cells	PSMA IHC vessels	PSMA PET	J591 imaging	Relevant uptake for therapy	Comments
					50% (1/2) showed at least one area of known metastatic disease targeted by ^{111}In-J591		
Siva, 2017 [61]	-	-	-	*Recurrence*<br>**N=1**<br>PET uptake: no	-	-	PET radiotracer: ^{68}Ga-PSMA
Yin, 2018 [68]	-	-	-	*Metastases*<br>**N=3**<br>PET uptake: 67% (2/3)<br>SUVmax: range: 1.8-4.1	-	-	PET radiotracer: [18F]DCFPyL
Raveenthiran, 2019 [64]	-	-	-	*Primary tumor*<br>**N=1**<br>PET uptake: no	-	-	PET radiotracer: ^{68}Ga-PSMA
**Subtype: undefined**							
Rhee, 2016 [54]	-	-	-	*Primary tumor*<br>**N=1**<br>PET uptake: yes<br>SUVmax: 18.3	-	-	PET radiotracer: ^{68}Ga-PSMA
Yin, 2018 [68]	-	-	-	*Metastases*<br>**N=2**<br>PET uptake: 50% (1/2)<br>SUVmax: range: 0.5-6.2	-	-	PET radiotracer: [18F]DCFPyL
Raveenthiran, 2019 [64]	-	-	-	*Primary tumor*<br>**N=6**<br>PET uptake: 67% (4/6)<br>SUVmax: NR	-	-	PET radiotracer: ^{68}Ga-PSMA
Mittlmeier, 2020 [69]	-	-	-	*Metastases*<br>**N=1**<br>PET uptake: yes	-	-	PET radiotracer: ^{18}F-PSMA-1007

Study	mRNA	PSMA IHC cells	PSMA IHC vessels	PSMA PET	J591 imaging	Relevant uptake for therapy	Comments
				SUVmax: NR			
**Hepatocellular carcinoma**							
Hirmas, 2021 [70]	-	-	-	*Primary tumor*<br>**N=39**<br>PET uptake: 97% (38/39)<br>Mean SUVmax: 16.2 (range: 5.9-55.4)<br><br>*Metastases*<br>**N=11**<br>PET uptake: 100% (11/11)<br>Mean SUVmax: NR (range: 2.9-21.3)	-		PET radiotracer: ^{68}Ga-PSMA
Jiao, 2019 [71]	-	*Primary tumor*<br>**N=103**<br>PSMA+: 0% (0/103)	*Primary tumor*<br>**N=103**<br>PSMA+: 74% (76/103)	-	-	-	
Kesler, 2018 [72]	-	*Primary tumor*<br>**N=5**<br>PSMA+: 0% (0/5)	*Primary tumor*<br>**N=5**<br>PSMA+: 60% (3/5)	*Primary tumor*<br>**N=37**<br>PET uptake: 97% (36/37)<br>median SUVmax: 11.7 (range: 3.7-41.2)<br><br>*Regenerative nodules*<br>**N=4**<br>PET uptake: 0% (0/4)<br>median SUVmax: NR	-	Tumors were divided in CT-enhancing and non-enhancing areas.<br>All SUVmax-TBR's of enhancing areas are ≥1.5.<br><br>Of the 10 non-enhancing areas, 4 have a SUVmax-TBR <1.5.	PET radiotracer: ^{68}Ga-PSMA<br><br>Added diagnostic value: identification of new extrahepatic metastases were found in 2 patients.

Study	mRNA	PSMA IHC cells	PSMA IHC vessels	PSMA PET	J591 imaging	Relevant uptake for therapy	Comments
Kuyumcu, 2019 [73]	-	-	-	*Primary tumor*<br>**N=19**<br>PET uptake: 84% (16/19)<br>Mean SUVmax: 17.4 (range: 3.8-36.9)<br><br>*Metastases*<br>**N=1**<br>PET uptake: yes<br>SUVmax: 4.3	-	Of all PSMA-PET-positive patients, 15/16 had a TBR ≥ 1.5 (range 1.5-9.7)	PET radiotracer: ^{68}Ga-PSMA
Tolkach, 2019 [74]	-	*Primary tumor*<br>N=168<br>PSMA+: 41% (69/168)<br>Canalicular patterns	*Primary tumor*<br>N=168<br>PSMA+: 90% (151/168)	*Primary tumor*<br>**N=1**<br>PET-uptake: yes<br>SUVmax: NR	-	-	PET radiotracer: ^{68}Ga-PSMA<br><br>Reports on mRNA expression (but is based on results of Cancer Genome Atlas)
Alipour, 2017 [75]	-	-	-	*Primary tumor*<br>**N=1**<br>PET uptake: yes<br>SUVmax: 9.9	-	-	PET radiotracer: ^{68}Ga-PSMA<br><br>Subtype hepatocellular cholangiocarcinoma
Das, 2020 [76]	-	-	-	*Primary tumor*<br>**N=1**<br>PET uptake: yes<br>SUVmax: 8.66	-	-	PET radiotracer: ^{68}Ga-PSMA
Erhamamci, 2020 [77]	-	-	-	*Primary tumor*<br>**N=1**<br>PET uptake: yes<br>SUVmax: 20.3	-	-	PET radiotracer: ^{68}Ga-PSMA
Patel, 2017 [78]	-	-	-	*Primary tumor*<br>**N=1**<br>PET uptake: yes<br>SUVmax: 9.1-11.2	-	-	PET radiotracer: ^{68}Ga-PSMA

Study	mRNA	PSMA IHC cells	PSMA IHC vessels	PSMA PET	J591 imaging	Relevant uptake for therapy	Comments
				Metastases<br>**N=2**<br>PET uptake: yes<br>SUVmax: NR			
Perez, 2019 [79]	-	-	-	*Primary tumor*<br>**N=1**<br>PET uptake: yes<br>SUVmax: 29.4	-	-	PET radiotracer: ^{68}Ga-PSMA
Taneja, 2017 [80]	-	-	-	*Primary tumor*<br>**N=1**<br>PET-uptake: yes<br>SUVmax: 15.7	-	-	PET radiotracer: ^{68}Ga-PSMA
Kunikowska, 2020 [81]	-	*Primary/local recurrence*<br>**N=6**<br>PSMA+: 0% (0/6)	*Primary/local recurrence*<br>**N=6**<br>PSMA+: 83% (5/6)	*Primary/local recurrence*<br>**N=15**<br>PET-uptake: 100% (15/15)<br>Mean SUVmax: 14.7 (range: 5.5-29.3)<br><br>*Metastases*<br>**N=2**<br>PET-uptake: 100% (2/2)<br>Mean SUVmax: 6.1 (range: 2.2-12.0)	-	100%; tumor/liver ratio >1.5 in 15/15 patients	PET radiotracer: ^{68}Ga-PSMA
**Lung cancer**							
**Subtype: non-small cell lung cancer (squamous cell carcinoma, adenocarcinoma, large cell carcinoma)**							
Liu, 1997 [82]	-	-	*Primary tumor*<br>**N=1**<br>PSMA+: yes	-	-	-	
Chang, 1999 [8]	-	*Primary tumor*<br>**N=5**<br>PSMA+: 0% (0/5)	*Primary tumor*<br>**N=5**<br>PSMA+: 100% (5/5)	-	-	-	

Study	mRNA	PSMA IHC cells	PSMA IHC vessels	PSMA PET	J591 imaging	Relevant uptake for therapy	Comments
Milowsky, 2007 [57]	-	-	-	-	*Metastases*<br>**N=3**<br>100% (3/3) showed at least one area of known metastatic disease targeted by ^{111}In-J591	-	
Wang, 2015 [83]	-	*Primary tumor*<br>SCC: **N=30**<br>PSMA+: 50% (15/30)<br><br>Adeno: **N=29**<br>PSMA+: 66% (19/29)<br><br>Large cell: **N=28**<br>PSMA+: 46% (13/28)	*Primary tumor*<br>SCC: **N=30**<br>PSMA+: 93% (28/30)<br><br>Adeno: **N=29**<br>PSMA+: 76% (22/29)<br><br>Large cell: **N=28**<br>PSMA+: 86% (24/28)	-	-	-	Of all NSCLC (N=87):<br>46% (40/87) expressed PSMA in tumor cells & 85% (74/87) expressed PSMA in neovasculature.
Pyka, 2016 [84]	-	-	*Primary tumor*<br>**N=7**<br>PSMA+: 100% (7/7)	*Primary tumor*<br>**N=7**<br>PET uptake: 100% (7/7)<br><u>Mean</u> SUVmax: 5.6 (range: NR)	-	-	PET radiotracer: ^{68}Ga-PSMA<br><br>Biased population: selected based on PSMA avid lesions on PET scan for prostate cancer.
Shetty, 2016 [85]	-	-	-	*Metastases*<br>**N=1**<br>PET uptake: yes<br>SUVmax: 4.4	-	-	PET radiotracer: ^{68}Ga-PSMA
Osman, 2017 [43]	-	-	-	*Primary tumor*<br>**N=2**	-	-	PET radiotracer: ^{68}Ga-PSMA

Study	mRNA	PSMA IHC cells	PSMA IHC vessels	PSMA PET	J591 imaging	Relevant uptake for therapy	Comments
				Median SUVmax: NR, range: 3.7-7.0			Biased population: selected based on PSMA avid lesions on PET scan for prostate cancer.
Schmidt, 2017 [86]	-	*Primary tumor*<br>SCC: **N=121**<br>PSMA+: 12% (14/121)<br><br>Adeno: **N=112**<br>PSMA+: 2% (2/112)<br><br>Large cell: **N=42**<br>PSMA+: 2% (1/42)	*Primary/metastases*<br>SCC: **N=121**<br>PSMA+: 57% (69/121)<br><br>Adeno: **N=112**<br>PSMA+: 37% (41/112)<br><br>Large cell: **N=42**<br>PSMA+: 60% (25/42)	-	-	-	
Chia, 2018 [87]	-	-	-	*Primary tumor*<br>**N=1** (adeno)<br>PET uptake: yes<br>SUVmax: 4.8	-	-	PET radiotracer: ^{68}Ga-PSMA
Jochumsen, 2018 [88]	-	-	-	*Primary tumor*<br>**N=1** (adeno)<br>PET uptake: yes<br>SUVmax: NR	-	-	PET radiotracer: ^{68}Ga-PSMA<br><br>The SUVmax was not reported but report states: “intense uptake”
Usmani, 2020 [89]	-	-	-	*Primary tumor*<br>**N=1** (adeno)<br>SUVmax: 5.6	-	-	
**Subtype: small cell lung cancer**							
Wang, 2015 [83]	-	*Primary tumor*<br>**N=30**<br>PSMA+: 0% (0/30)	*Primary tumor*<br>**N=30**<br>PSMA+: 70% (21/30)	-	-	-	
**Breast cancer**							
**Subtype: ductal carcinoma**							

Study	mRNA	PSMA IHC cells	PSMA IHC vessels	PSMA PET	J591 imaging	Relevant uptake for therapy	Comments
Chang, 1999 [8]	-	*Primary tumor*<br>**N=5**<br>PSMA+: 0% (0/5)<br>median positive cells: NR	*Primary tumor*<br>**N=5**<br>PSMA+: 100% (5/5)	-	-	-	
Kinoshita, 2006 [47]	-	-	*Primary tumor*§<br>**N=5**<br>PSMA+: 12.5% (1/5)	-	-	-	§Study did not mention if PSMA expression was observed in tumor cells or neovasculature.
Kasoha, 2017 [90]	-	*Primary tumor*<br>**N=51**<br>PSMA+: 51% (26/51)	*Primary tumor*<br>**N=51**<br>PSMA+: 80% (41/51)	-	-	-	
Tolkach 2018 [91]	-	-	*Primary tumor:*<br>**N=251**<br>PSMA+: 65% (162/251)	-	-	-	
Kumar, 2018 [92]	-	-	-	*Primary tumor:*<br>**N=1**<br>PET uptake: yes<br>SUVmax: 9.7<br><br>*Lymph node*:<br>**N=1**<br>PET uptake: yes<br>SUVmax: 6.5	-	-	PET radiotracer: ^{68}Ga-PSMA<br><br>In male, BRCA 2 mutation.
Polverari, 2019 [93]	-	-	-	*Primary tumor:*<br>**N=1**<br>PET uptake: yes<br>SUVmax: 3.2	-	-	PET radiotracer: ^{68}Ga-PSMA<br><br>In male, BRCA 2 mutation. Also diagnosed with

Study	mRNA	PSMA IHC cells	PSMA IHC vessels	PSMA PET	J591 imaging	Relevant uptake for therapy	Comments
							prostate cancer, including pelvic nodal metastasis.
**Subtype: lobular carcinoma**							
Chang, 1999 [8]	-	*Primary tumor*<br>**N=1**<br>PSMA+: 0% (0/1)<br>median positive cells: NR	*Primary tumor*<br>**N=1**<br>PSMA+: 0% (0/1)	-	-	-	
Tolkach, 2018 [91]	-	-	*Primary tumor:*<br>**N=64**<br>PSMA+: 42% (27/64)	-	-	-	
**Subtype: undefined**							
Liu, 1997 [82]	-	-	*Primary tumor*<br>**N=1**<br>PSMA+: yes (1/1)	-	-	-	
Wernicke, 2014 [94]	-	-	*Primary tumor*¶<br>**N=92**<br>PSMA+: 74% (68/92)<br><br>*Metastases*¶<br>**N=14**<br>PSMA+: 100% (14/14)	-	-	-	¶ subtypes: 70% IDC, 20% DCIS, 10% ILC. Results were not reported per subtype, only for total cohort.
Kasoha, 2017 [90]	-	*Primary tumor#*<br>**N=17**<br>PSMA+: 29% (5/17)<br><br>*Metastases***<br>**N=12**<br>PSMA+: 75% (9/12)	*Primary tumor#*<br>**N=17**<br>PSMA+: 47% (8/17)<br><br>*Metastases***<br>**N=9**<br>PSMA+: 89% (8/9)	-	-	-	# subtypes: ILC: N=12, ILD/IDC: N=3, tubular carcinoma: N=1, mucinous carcinoma N=1.

Study	mRNA	PSMA IHC cells	PSMA IHC vessels	PSMA PET	J591 imaging	Relevant uptake for therapy	Comments
							Results were not reported per subtype, only for total cohort.<br><br>** report does not specify the subtypes of patients with metastases.
Sathekge, 2017 [95]	-	-	-	*Primary/local recurrence*<br>**N=13**<br>PET uptake: 54% (7/13)<br>Mean SUVmax: 2.45 (SD: 2.55)<br><br>*Metastases*<br>**N=14**<br>PET uptake: 86% (12/14)<br>Mean SUVmax: 6.86 (SD 5.68)	**-**	**-**	PET radiotracer: ^{68}Ga-PSMA<br><br>Cohort consisted of: IDC: N=13, ILC: N=2, neuro-endocrine: N=1, unclear: N=3<br><br>Overall 81 tumor lesions on routine examination, 84% detection rate on ^{68}Ga-PSMA PET.
Sathekge, 2015 [96]	-	-	-	*Metastases*<br>**N=1**<br>PET uptake: yes<br>SUVmax: NR	**-**	**-**	PET radiotracer: ^{68}Ga-PSMA
Malik, 2018 [97]	-	-	-	*Metastases*<br>**N=1**<br>PET uptake: yes<br>SUVmax: NR	**-**	**-**	PET radiotracer: ^{68}Ga-PSMA
Passah, 2018 [98]	-	-	-	*Metastases*<br>**N=1**<br>PET uptake: yes<br>SUVmax: NR	**-**	Uptake in metastases was slightly more than liver.	PET radiotracer: ^{68}Ga-PSMA
Marafi, 2020 [99]	-	-	-	*Metastases*<br>**N=1**	**-**	-	PET radiotracer: ^{18}F-PSMA

Study	mRNA	PSMA IHC cells	PSMA IHC vessels	PSMA PET	J591 imaging	Relevant uptake for therapy	Comments
				PET uptake: yes<br>SUVmax: NR			
Milowsky, 2007 [57]	-	-	-	-	*Metastases*<br>**N=3**<br>67% (2/3) showed at least one area of known metastatic disease targeted by ^{111}In-J591	-	
Marafi, 2020 [100]	-	-	-	*Primary/local recurrence*<br>**N=1**<br>PET uptake: yes<br>SUVmax: 5.0<br><br>*Metastases*<br>**N=1**<br>PET uptake: yes<br>SUVmax: NR	-	-	PET radiotracer: ^{18}F-PSMA

Abbreviations

IHC: immunohistochemistry, IQR: inter quartile range, NR: not reported, N: number of patients, SUV: Maximum standardized uptake value, PSMA: prostate specific membrane antigen, ^{68}Ga: Gallium-68, TBR: tumor-to-background ratio, SD: standard deviation, IQR: interquartile range.

References

[1] Klein Nulent TJW, Valstar MH, Smit LA, Smeele LE, Zuithoff NPA, de Keizer B, et al. Prostate-specific membrane antigen (PSMA) expression in adenoid cystic carcinoma of the head and neck. BMC Cancer. 2020;20:519.
[2] van Boxtel W, Lutje S, van Engen-van Grunsven ICH, Verhaegh GW, Schalken JA, Jonker MA, et al. (68)Ga-PSMA-HBED-CC PET/CT imaging for adenoid cystic carcinoma and salivary duct carcinoma: a phase 2 imaging study. Theranostics. 2020;10:2273-83.
[3] Klein Nulent TJW, van Es RJJ, Krijger GC, de Bree R, Willems SM, de Keizer B. Prostate-specific membrane antigen PET imaging and immunohistochemistry in adenoid cystic carcinoma-a preliminary analysis. Eur J Nucl Med Mol Imaging. 2017;44:1614-21.
[4] Konig L, Hauswald H, Flechtenmacher C, Heller M, Debus J, Haberkorn U, et al. Uptake of Prostate-Specific Membrane Antigen (PSMA) in adenoid cystic carcinoma - Is PSMA-PET-CT a helpful tool in radiation oncology? Clin Transl Radiat Oncol. 2017;7:79-82.
[5] de Keizer B, Krijger GC, Ververs FT, van Es RJJ, de Bree R, Willems S. (68)Ga-PSMA PET-CT Imaging of Metastatic Adenoid Cystic Carcinoma. Nucl Med Mol Imaging. 2017;51:360-1.
[6] Lutje S, Sauerwein W, Lauenstein T, Bockisch A, Poeppel TD. In Vivo Visualization of Prostate-Specific Membrane Antigen in Adenoid Cystic Carcinoma of the Salivary Gland. Clin Nucl Med. 2016;41:476-7.
[7] Has Simsek D, Kuyumcu S, Agaoglu FY, Unal SN. Radionuclide Therapy With 177Lu-PSMA in a Case of Metastatic Adenoid Cystic Carcinoma of the Parotid. Clin Nucl Med. 2019;44:764-6.
[8] Chang SS, Reuter VE, Heston WD, Bander NH, Grauer LS, Gaudin PB. Five different anti-prostate-specific membrane antigen (PSMA) antibodies confirm PSMA expression in tumor-associated neovasculature. Cancer Res. 1999;59:3192-8.
[9] Salas Fragomeni RA, Menke JR, Holdhoff M, Ferrigno C, Laterra JJ, Solnes LB, et al. Prostate-Specific Membrane Antigen-Targeted Imaging With [18F]DCFPyL in High-Grade Gliomas. Clin Nucl Med. 2017;42:e433-e5.
[10] Nomura N, Pastorino S, Jiang P, Lambert G, Crawford JR, Gymnopoulos M, et al. Prostate specific membrane antigen (PSMA) expression in primary gliomas and breast cancer brain metastases. Cancer Cell Int. 2014;14:26.
[11] Wernicke AG, Edgar MA, Lavi E, Liu H, Salerno P, Bander NH, et al. Prostate-specific membrane antigen as a potential novel vascular target for treatment of glioblastoma multiforme. Arch Pathol Lab Med. 2011;135:1486-9.
[12] Schwenck J, Tabatabai G, Skardelly M, Reischl G, Beschorner R, Pichler B, et al. In vivo visualization of prostate-specific membrane antigen in glioblastoma. Eur J Nucl Med Mol Imaging. 2015;42:170-1.
[13] Saffar H, Noohi M, Tavangar SM, Saffar H, Azimi S. Expression of Prostate-Specific Membrane Antigen (PSMA) in Brain Glioma and its Correlation with Tumor Grade. Iran J Pathol. 2018;13:45-53.
[14] Mahzouni P, Shavakhi M. Prostate-Specific Membrane Antigen Expression in Neovasculature of Glioblastoma Multiforme. Adv Biomed Res. 2019;8:18.
[15] Sasikumar A, Joy A, Pillai MR, Nanabala R, Anees KM, Jayaprakash PG, et al. Diagnostic Value of 68Ga PSMA-11 PET/CT Imaging of Brain Tumors-Preliminary Analysis. Clin Nucl Med. 2017;42:e41-e8.
[16] Kunikowska J, Bartosz K, Leszek K. Glioblastoma multiforme: another potential application for (68)Ga-PSMA PET/CT as a guide for targeted therapy. Eur J Nucl Med Mol Imaging. 2018;45:886-7.
[17] Sasikumar A, Kashyap R, Joy A, Charan Patro K, Bhattacharya P, Reddy Pilaka VK, et al. Utility of 68Ga-PSMA-11 PET/CT in Imaging of Glioma-A Pilot Study. Clin Nucl Med. 2018;43:e304-e9.
[18] Verma P, Malhotra G, Goel A, Rakshit S, Chandak A, Chedda R, et al. Differential Uptake of 68Ga-PSMA-HBED-CC (PSMA-11) in Low-Grade Versus High-Grade Gliomas in Treatment-Naive Patients. Clin Nucl Med. 2019;44:e318-e22.

[19] Gupta M, Choudhury PS, Premsagar IC, Gairola M, Ahlawat P. Role of 68Ga-Prostate-Specific Membrane Antigen PET/CT in Disease Assessment in Glioblastoma Within 48 Hours of Surgery. Clin Nucl Med. 2020;45:204-5.
[20] Kunikowska J, Charzynska I, Kulinski R, Pawlak D, Maurin M, Krolicki L. Tumor uptake in glioblastoma multiforme after IV injection of [(177)Lu]Lu-PSMA-617. Eur J Nucl Med Mol Imaging. 2020.
[21] Kunikowska J, Kulinski R, Muylle K, Koziara H, Krolicki L. 68Ga-Prostate-Specific Membrane Antigen-11 PET/CT: A New Imaging Option for Recurrent Glioblastoma Multiforme? Clin Nucl Med. 2020;45:11-8.
[22] Marafi F, Sasikumar A, Fathallah W, Esmail A. 18F-PSMA 1007 Brain PET/CT Imaging in Glioma Recurrence. Clin Nucl Med. 2020;45:e61-e2.
[23] Pilati E, Nicolotti DG, Ceci F, Finessi M, Cerio I, Dionisi B, et al. 68Ga-Prostate-Specific Membrane Antigen 11 PET/CT Detects Residual Glioblastoma After Radical Surgery in a Patient With Synchronous Recurrent Prostate Cancer: A Case Report. Clin Nucl Med. 2020;45:e151-e3.
[24] Kumar A, Ballal S, Yadav MP, ArunRaj ST, Haresh KP, Gupta S, et al. 177Lu-/68Ga-PSMA Theranostics in Recurrent Glioblastoma Multiforme: Proof of Concept. Clin Nucl Med. 2020.
[25] Ciappuccini R, Edet-Sanson A, Saguet-Rysanek V, Gauthe M, Bardet S. Thyroid Incidentaloma on 18F-fluorocholine PET/CT and 68Ga-PSMA PET/CT Revealing a Medullary Thyroid Carcinoma. Clin Nucl Med. 2019;44:663-5.
[26] Arora S, Damle NA, Parida GK, Singhal A, Nalli H, Dattagupta S, et al. Recurrent Medullary Thyroid Carcinoma on 68Ga-Prostate-Specific Membrane Antigen PET/CT: Exploring New Theranostic Avenues. Clin Nucl Med. 2018;43:359-60.
[27] Arora S, Prabhu M, Damle NA, Bal C, Kumar P, Nalla H, et al. Prostate-specific Membrane Antigen Imaging in Recurrent Medullary Thyroid Cancer: A New Theranostic Tracer in the Offing? Indian J Nucl Med. 2018;33:261-3.
[28] Bychkov A, Vutrapongwatana U, Tepmongkol S, Keelawat S. PSMA expression by microvasculature of thyroid tumors - Potential implications for PSMA theranostics. Sci Rep. 2017;7:5202.
[29] Heitkotter B, Steinestel K, Trautmann M, Grunewald I, Barth P, Gevensleben H, et al. Neovascular PSMA expression is a common feature in malignant neoplasms of the thyroid. Oncotarget. 2018;9:9867-74.
[30] Lodewijk L, Willems SM, Dreijerink KMA, de Keizer B, van Diest PJ, Schepers A, et al. The theranostic target prostate-specific membrane antigen is expressed in medullary thyroid cancer. Hum Pathol. 2018;81:245-54.
[31] Damle NA, Bal C, Singh TP, Gupta R, Reddy S, Kumar R, et al. Anaplastic thyroid carcinoma on 68 Ga-PSMA PET/CT: opening new frontiers. Eur J Nucl Med Mol Imaging. 2018;45:667-8.
[32] Moore M, Panjwani S, Mathew R, Crowley M, Liu YF, Aronova A, et al. Well-Differentiated Thyroid Cancer Neovasculature Expresses Prostate-Specific Membrane Antigen-a Possible Novel Therapeutic Target. Endocr Pathol. 2017;28:339-44.
[33] Lawhn-Heath C, Yom SS, Liu C, Villanueva-Meyer JE, Aslam M, Smith R, et al. Gallium-68 prostate-specific membrane antigen ([(68)Ga]Ga-PSMA-11) PET for imaging of thyroid cancer: a feasibility study. EJNMMI Res. 2020;10:128.
[34] de Vries LH, Lodewijk L, Braat A, Krijger GC, Valk GD, Lam M, et al. (68)Ga-PSMA PET/CT in radioactive iodine-refractory differentiated thyroid cancer and first treatment results with (177)Lu-PSMA-617. EJNMMI Res. 2020;10:18.
[35] Lengana T, Lawal IO, Mokoala K, Vorster M, Sathekge MM. 68Ga-PSMA: a One-stop Shop in Radioactive Iodine Refractory Thyroid Cancer? Nucl Med Mol Imaging. 2019;53:442-5.
[36] Singh D, Horneman R, Nagra NK. More than the prostate: Intrapancreatic accessory spleen and papillary thyroid cancer detected with (18)F-PSMA PET/CT. Hell J Nucl Med. 2018;21:145-7.
[37] Lutje S, Gomez B, Cohnen J, Umutlu L, Gotthardt M, Poeppel TD, et al. Imaging of Prostate-Specific Membrane Antigen Expression in Metastatic Differentiated Thyroid Cancer Using 68Ga-HBED-CC-PSMA PET/CT. Clin Nucl Med. 2017;42:20-5.
[38] Sager S, Vatankulu B, Uslu L, Sonmezoglu K. Incidental Detection of Follicular Thyroid Carcinoma in 68Ga-PSMA PET/CT Imaging. J Nucl Med Technol. 2016;44:199-200.

[39] Verma P, Malhotra G, Agrawal R, Sonavane S, Meshram V, Asopa RV. Evidence of Prostate-Specific Membrane Antigen Expression in Metastatic Differentiated Thyroid Cancer Using 68Ga-PSMA-HBED-CC PET/CT. Clin Nucl Med. 2018;43:e265-e8.
[40] Sasikumar A, Joy A, Pillai MRA, Oommen KE, Jayakumar R. Rare Case of Intratracheal Metastasis Detected on 68Ga-Prostate-Specific Membrane Antigen PET/CT Scan in a Case of Thyroglobulin Elevated Negative Iodine Scan Syndrome. Clin Nucl Med. 2018;43:282-3.
[41] Taywade SK, Damle NA, Bal C. PSMA Expression in Papillary Thyroid Carcinoma: Opening a New Horizon in Management of Thyroid Cancer? Clin Nucl Med. 2016;41:e263-5.
[42] Verburg FA, Krohn T, Heinzel A, Mottaghy FM, Behrendt FF. First evidence of PSMA expression in differentiated thyroid cancer using [(6)(8)Ga]PSMA-HBED-CC PET/CT. Eur J Nucl Med Mol Imaging. 2015;42:1622-3.
[43] Osman MM, Iravani A, Hicks RJ, Hofman MS. Detection of Synchronous Primary Malignancies with (68)Ga-Labeled Prostate-Specific Membrane Antigen PET/CT in Patients with Prostate Cancer: Frequency in 764 Patients. J Nucl Med. 2017;58:1938-42.
[44] Sollini M, di Tommaso L, Kirienko M, Piombo C, Erreni M, Lania AG, et al. PSMA expression level predicts differentiated thyroid cancer aggressiveness and patient outcome. EJNMMI Res. 2019;9:93.
[45] Joshi A, Nicholson C, Rhee H, Gustafson S, Miles K, Vela I. Incidental Malignancies Identified During Staging for Prostate Cancer With (68)Ga Prostate-specific Membrane Antigen HBED-CC Positron Emission Tomography Imaging. Urology. 2017;104:e3-e4.
[46] Chang SS, Reuter VE, Heston WD, Gaudin PB. Metastatic renal cell carcinoma neovasculature expresses prostate-specific membrane antigen. Urology. 2001;57:801-5.
[47] Kinoshita Y, Kuratsukuri K, Landas S, Imaida K, Rovito PM, Jr., Wang CY, et al. Expression of prostate-specific membrane antigen in normal and malignant human tissues. World J Surg. 2006;30:628-36.
[48] Baccala A, Sercia L, Li J, Heston W, Zhou M. Expression of prostate-specific membrane antigen in tumor-associated neovasculature of renal neoplasms. Urology. 2007;70:385-90.
[49] Al-Ahmadie HA, Olgac S, Gregor PD, Tickoo SK, Fine SW, Kondagunta GV, et al. Expression of prostate-specific membrane antigen in renal cortical tumors. Mod Pathol. 2008;21:727-32.
[50] Spatz S, Tolkach Y, Jung K, Stephan C, Busch J, Ralla B, et al. Comprehensive Evaluation of Prostate Specific Membrane Antigen Expression in the Vasculature of Renal Tumors: Implications for Imaging Studies and Prognostic Role. J Urol. 2018;199:370-7.
[51] Demirci E, Ocak M, Kabasakal L, Decristoforo C, Talat Z, Halac M, et al. (68)Ga-PSMA PET/CT imaging of metastatic clear cell renal cell carcinoma. Eur J Nucl Med Mol Imaging. 2014;41:1461-2.
[52] Rowe SP, Gorin MA, Hammers HJ, Som Javadi M, Hawasli H, Szabo Z, et al. Imaging of metastatic clear cell renal cell carcinoma with PSMA-targeted (1)(8)F-DCFPyL PET/CT. Ann Nucl Med. 2015;29:877-82.
[53] Rowe SP, Gorin MA, Hammers HJ, Pomper MG, Allaf ME, Javadi MS. Detection of 18F-FDG PET/CT Occult Lesions With 18F-DCFPyL PET/CT in a Patient With Metastatic Renal Cell Carcinoma. Clin Nucl Med. 2016;41:83-5.
[54] Rhee H, Blazak J, Tham CM, Ng KL, Shepherd B, Lawson M, et al. Pilot study: use of gallium-68 PSMA PET for detection of metastatic lesions in patients with renal tumour. EJNMMI Res. 2016;6:76.
[55] Meyer AR, Carducci MA, Denmeade SR, Markowski MC, Pomper MG, Pierorazio PM, et al. Improved identification of patients with oligometastatic clear cell renal cell carcinoma with PSMA-targeted (18)F-DCFPyL PET/CT. Ann Nucl Med. 2019;33:617-23.
[56] Sawicki LM, Buchbender C, Boos J, Giessing M, Ermert J, Antke C, et al. Diagnostic potential of PET/CT using a (68)Ga-labelled prostate-specific membrane antigen ligand in whole-body staging of renal cell carcinoma: initial experience. Eur J Nucl Med Mol Imaging. 2017;44:102-7.
[57] Milowsky MI, Nanus DM, Kostakoglu L, Sheehan CE, Vallabhajosula S, Goldsmith SJ, et al. Vascular targeted therapy with anti-prostate-specific membrane antigen monoclonal antibody J591 in advanced solid tumors. J Clin Oncol. 2007;25:540-7.

[58] Einspieler I, Tauber R, Maurer T, Schwaiger M, Eiber M. 68Ga Prostate-Specific Membrane Antigen Uptake in Renal Cell Cancer Lymph Node Metastases. Clin Nucl Med. 2016;41:e261-2.
[59] Rhee H, Ng KL, Tse BW, Yeh MC, Russell PJ, Nelson C, et al. Using prostate specific membrane antigen (PSMA) expression in clear cell renal cell carcinoma for imaging advanced disease. Pathology. 2016;48:613-6.
[60] Sasikumar A, Joy A, Nanabala R, Unni M, Tk P. Complimentary Pattern of Uptake in 18F-FDG PET/CT and 68Ga-Prostate-Specific Membrane Antigen PET/CT in a Case of Metastatic Clear Cell Renal Carcinoma. Clin Nucl Med. 2016;41:e517-e9.
[61] Siva S, Callahan J, Pryor D, Martin J, Lawrentschuk N, Hofman MS. Utility of (68) Ga prostate specific membrane antigen - positron emission tomography in diagnosis and response assessment of recurrent renal cell carcinoma. J Med Imaging Radiat Oncol. 2017;61:372-8.
[62] Zacho HD, Nielsen JB, Dettmann K, Haberkorn U, Petersen LJ. Incidental Detection of Thyroid Metastases From Renal Cell Carcinoma Using 68Ga-PSMA PET/CT to Assess Prostate Cancer Recurrence. Clin Nucl Med. 2017;42:221-2.
[63] Gao J, Xu Q, Fu Y, He K, Zhang C, Zhang Q, et al. Comprehensive evaluation of (68)Ga-PSMA-11 PET/CT parameters for discriminating pathological characteristics in primary clear-cell renal cell carcinoma. Eur J Nucl Med Mol Imaging. 2020.
[64] Raveenthiran S, Esler R, Yaxley J, Kyle S. The use of (68)Ga-PET/CT PSMA in the staging of primary and suspected recurrent renal cell carcinoma. Eur J Nucl Med Mol Imaging. 2019;46:2280-8.
[65] Mittlmeier LM, Unterrainer M, Todica A, Cyran CC, Rodler S, Bartenstein P, et al. PSMA PET/CT for tyrosine-kinase inhibitor monitoring in metastatic renal cell carcinoma. Eur J Nucl Med Mol Imaging. 2020;47:2216-7.
[66] Marafi F, Sasikumar A, Al-Terki A, Alfeeli M. 18F-PSMA 1007 in Suspected Renal Cell Carcinoma. Clin Nucl Med. 2020;45:377-8.
[67] Chen EJ, Tan TH, Chew MT, Chye PC. 68Ga-PSMA PET/CT and 18F-FDG PET/CT in Renal Cell Carcinoma. Clin Nucl Med. 2020;45:e317-e9.
[68] Yin Y, Campbell SP, Markowski MC, Pierorazio PM, Pomper MG, Allaf ME, et al. Inconsistent Detection of Sites of Metastatic Non-Clear Cell Renal Cell Carcinoma with PSMA-Targeted [(18)F]DCFPyL PET/CT. Mol Imaging Biol. 2019;21:567-73.
[69] Mittlmeier LM, Unterrainer M, Todica A, Cyran CC, Rodler S, Bartenstein P, et al. PSMA PET/CT for tyrosine-kinase inhibitor monitoring in metastatic renal cell carcinoma. Eur J Nucl Med Mol Imaging. 2020.
[70] Hirmas N, Leyh C, Sraieb M, Barbato F, Schaarschmidt BM, Umutlu L, et al. [(68)Ga]Ga-PSMA-11 PET/CT improves tumor detection and impacts management in patients with hepatocellular carcinoma (HCC). J Nucl Med. 2021.
[71] Jiao D, Li Y, Yang F, Han D, Wu J, Shi S, et al. Expression of Prostate-Specific Membrane Antigen in Tumor-Associated Vasculature Predicts Poor Prognosis in Hepatocellular Carcinoma. Clin Transl Gastroenterol. 2019;10:1-7.
[72] Kesler M, Levine C, Hershkovitz D, Mishani E, Menachem Y, Lerman H, et al. (68)Ga-PSMA is a novel PET-CT tracer for imaging of hepatocellular carcinoma: A prospective pilot study. J Nucl Med. 2018.
[73] Kuyumcu S, Has-Simsek D, Iliaz R, Sanli Y, Buyukkaya F, Akyuz F, et al. Evidence of Prostate-Specific Membrane Antigen Expression in Hepatocellular Carcinoma Using 68Ga-PSMA PET/CT. Clin Nucl Med. 2019;44:702-6.
[74] Tolkach Y, Goltz D, Kremer A, Ahmadzadehfar H, Bergheim D, Essler M, et al. Prostate-specific membrane antigen expression in hepatocellular carcinoma: potential use for prognosis and diagnostic imaging. Oncotarget. 2019;10:4149-60.
[75] Alipour R, Gupta S, Trethewey S. Ga-68-PSMA Uptake in Combined Hepatocellular Cholangiocarcinoma With Skeletal Metastases. Clinical Nuclear Medicine. 2017;42:E452-E3.
[76] Das J, Ray S, Tapadia R, Midha D, Mallick I. Prostate-specific membrane antigen-expressing hepatic lesion: Metastatic or hepatocellular carcinoma. Indian Journal of Nuclear Medicine. 2020;35:58-60.
[77] Erhamamci S, Aslan N. Primary Hepatocellular Carcinoma With Intense Ga-68-PSMA Uptake But Slight F-18-FDG Uptake on PET/CT Imaging. Clinical Nuclear Medicine. 2020;45:E176-E7.

[78] Patel D, Loh H, Le K, Stevanovic A, Mansberg R. Incidental Detection of Hepatocellular Carcinoma on Ga-68-Labeled Prostate-Specific Membrane Antigen PET/CT. Clinical Nuclear Medicine. 2017;42:881-4.
[79] Perez PM, Flavell RR, Kelley RK, Umetsu S, Behr SC. Heterogeneous Uptake of F-18-FDG and Ga-68-PSMA-11 in Hepatocellular Carcinoma. Clinical Nuclear Medicine. 2019;44:e133-e5.
[80] Taneja S, Taneja R, Kashyap V, Jha A, Jena A. Ga-68-PSMA Uptake in Hepatocellular Carcinoma. Clinical Nuclear Medicine. 2017;42:E69-E70.
[81] Kunikowska J, Cieślak B, Gierej B, Patkowski W, Kraj L, Kotulski M, et al. [(68) Ga]Ga-Prostate-Specific Membrane Antigen PET/CT: a novel method for imaging patients with hepatocellular carcinoma. Eur J Nucl Med Mol Imaging. 2020.
[82] Liu H, Moy P, Kim S, Xia Y, Rajasekaran A, Navarro V, et al. Monoclonal antibodies to the extracellular domain of prostate-specific membrane antigen also react with tumor vascular endothelium. Cancer Res. 1997;57:3629-34.
[83] Wang HL, Wang SS, Song WH, Pan Y, Yu HP, Si TG, et al. Expression of prostate-specific membrane antigen in lung cancer cells and tumor neovasculature endothelial cells and its clinical significance. PLoS One. 2015;10:e0125924.
[84] Pyka T, Weirich G, Einspieler I, Maurer T, Theisen J, Hatzichristodoulou G, et al. 68Ga-PSMA-HBED-CC PET for Differential Diagnosis of Suggestive Lung Lesions in Patients with Prostate Cancer. J Nucl Med. 2016;57:367-71.
[85] Shetty D, Loh H, Bui C, Mansberg R, Stevanovic A. Elevated 68Ga Prostate-Specific Membrane Antigen Activity in Metastatic Non-Small Cell Lung Cancer. Clin Nucl Med. 2016;41:414-6.
[86] Schmidt LH, Heitkötter B, Schulze AB, Schliemann C, Steinestel K, Trautmann M, et al. Prostate specific membrane antigen (PSMA) expression in non-small cell lung cancer. PLoS One. 2017;12:e0186280.
[87] Chia JY, Loi HY, Khor LK, Joe Lee KC, Seow YH. Primary Lung Adenocarcinoma With (68)Gallium Prostate-Specific Membrane Antigen-PET/CT Scan Avidity in a Patient on Surveillance After Prostatectomy. Clin Genitourin Cancer. 2018;16:e525-e7.
[88] Jochumsen MR, Gormsen LC, Nielsen GL. 68Ga-PSMA Avid Primary Adenocarcinoma of the Lung With Complementary Low 18F-FDG Uptake. Clin Nucl Med. 2018;43:117-9.
[89] Usmani S, Sadeq A, Marafi F, Esmail A, Al Kandari F, Ahmed N. 68Ga-PMSA Uptake in the Lung: Metastatic Versus Primary Lung Tumor. Clin Nucl Med. 2020;45:e80-e2.
[90] Kasoha M, Unger C, Solomayer E-F, Bohle RM, Zaharia C, Khreich F, et al. Prostate-specific membrane antigen (PSMA) expression in breast cancer and its metastases. Clinical & experimental metastasis. 2017;34:479-90.
[91] Tolkach Y, Gevensleben H, Bundschuh R, Koyun A, Huber D, Kehrer C, et al. Prostate-specific membrane antigen in breast cancer: a comprehensive evaluation of expression and a case report of radionuclide therapy. Breast cancer research and treatment. 2018;169:447-55.
[92] Kumar R, Mittal BR, Bhattacharya A, Singh H, Singh SK. Synchronous Detection of Male Breast Cancer and Prostatic Cancer in a Patient With Suspected Prostatic Carcinoma on 68Ga-PSMA PET/CT Imaging. 2018. p. 431-2.
[93] Polverari G, Ceci F, Calderoni L, Cervati V, Farolfi A, Castellucci P, et al. Male Breast Cancer Detected by 68Ga-PSMA-11 PET/CT in a Patient With Prostate Cancer With Pelvic Lymph Node Metastasis. 2019. p. 154-6.
[94] Wernicke AG, Varma S, Greenwood EA, Christos PJ, Chao KSC, Liu H, et al. Prostate-specific membrane antigen expression in tumor-associated vasculature of breast cancers. APMIS : acta pathologica, microbiologica, et immunologica Scandinavica. 2014;122:482-9.
[95] Sathekge M, Lengana T, Modiselle M, Vorster M, Zeevaart J, Maes A, et al. 68Ga-PSMA-HBED-CC PET imaging in breast carcinoma patients2017.
[96] Sathekge M, Modiselle M, Vorster M, Mokgoro N, Nyakale N, Mokaleng B, et al. ^{68}Ga-PSMA imaging of metastatic breast cancer. 2015. p. 1482-3.
[97] Malik D, Kumar R, Mittal BR, Singh H, Bhattacharya A, Singh SK. 68Ga-Labeled PSMA Uptake in Nonprostatic Malignancies: Has the Time Come to Remove "PS" From PSMA? 2018. p. 529-32.
[98] Passah A, Arora S, Damle NA, Tripathi M, Bal C, Subudhi TK, et al. 68Ga-Prostate-Specific Membrane Antigen PET/CT in Triple-Negative Breast Cancer. 2018. p. 460-1.
[99] Marafi F, Sasikumar A, Alfeeli M, Fathallah W. 18F-PSMA 1007 Uptake in Brain Metastases From Breast Cancer. 2020. p. e77-e9.

[100] Marafi F, Sasikumar A, Alfeeli M, Thuruthel S. 18F-PSMA 1007 Uptake in a Man With Metastatic Breast Cancer. Clin Nucl Med. 2020;45:e276-e8.
